# Heat transfer enhancement of a solar air heater using capsule-shaped turbulators: a numerical analysis

**DOI:** 10.1038/s41598-025-99294-0

**Published:** 2025-05-02

**Authors:** N. Madhwesh, K. Vasudeva Karanth, Shiva Kumar

**Affiliations:** Department of Mechanical and Industrial Engineering, Manipal Institute of Technology, Manipal Academy of Higher Education, Manipal, Karnataka 576104 India

**Keywords:** Solar air heater, Turbulator, Orientation angle, Pitch ratio, Height ratio, Reynolds number, Nusselt number, Exergy efficiency, Energy science and technology, Engineering

## Abstract

The development of a laminar sublayer inside the channel of the solar collector results in a poor heat transfer coefficient. Adding artificial roughness to the absorber plate in the form of turbulators is an effective and promising method of increasing heat transmission by interrupting the laminar sublayer. In this study, a capsule-shaped turbulator is used to enhance the thermal performance of the solar air heater. With flow Re (Reynolds number) fluctuating from 3000 to 21,000, the flow and heat transfer-related parameters of an air heater fitted with turbulators were numerically examined. The orientation angle of the capsule turbulator corresponding to the flow direction is modified from 0° to 90° in steps of 15°. The outcomes indicated that the overall thermal performance of the solar collector was optimized for the orientation angle of 45° at all the Reynolds numbers chosen for the study. Beyond this angle, the turbulators block the flow, increasing the pumping power. The number of rows of turbulator, pitch and height ratios of the optimized turbulator design is varied in the range of 1–3, 0.025–0.25 and 0.004–0.008, respectively, to determine their optimum values for better performance. It is observed that the turbulator having two rows with a pitch ratio of 0.05 and height ratio of 0.006 produces a relatively higher overall thermal enhancement by about 15.76% when compared with the base model without turbulators. The same configuration yields about 52.64% improvement in the exergy efficiency with respect to the base model.

## Introduction

For quality and sustainable living, energy is necessary in many ways in our daily lives. The utilization of non-conventional and renewable energy sources has significantly increased because of the diminution of traditional energy sources and the rising need for energy for industrialization and economic growth. Solar energy is regarded as the most significant and hopeful energy source in the modern world.

Flat plate-type sun collectors are usually adequate for basic solar air heating applications. The laminar viscous layer formed by air movement inside the solar air heater is attached to the absorbing plate. It opposes heat transmission between the plate and the surrounding contacting fluid. The air heater performs poorly since the absorber air has a low convective heat transfer coefficient (h). Numerous studies have been done to boost heat transfer by eliminating the viscous sublayer by employing turbulence-creating elements on the bottom of absorber plates. The laminar layer linked to the absorber plate is disturbed by an artificially roughened absorber plate, which lowers heat resistance. As a result, adding roughness to the absorber plate is considered one of the most effective ways to improve the performance. However, the pressure drop across the flow path will increase. Such flow disruptions require energy to be produced, which comes from the blower and increases power requirements. To create an effective system, it is crucial to research solar heating systems’ thermal and hydraulic performances. Thermohydraulic enhancement factor (THEF) links hydraulic-related parameters, such as frictional parameters and pressure losses in the duct, with the performance of the heat transfer process inside the system.

Several scholars have examined the consequences of various turbulator forms on the effectiveness of solar air heaters. The influence of artificial roughness on the thermal–hydraulic characteristics of SAHs has been comprehensively investigated. Partha Pratim et al.^1^ altered a flat absorber plate with square barriers and threaded pin fins to enhance the performance. They determined that using the redesigned absorber plate increased the solar air heater’s exit temperature by about 9%. Abuska^2^ described a conical-surfaced absorber plate design by considering three mass flow rates. Findings were compared to those of a standard case. The results reveal that the thermal efficiency of the modified case is higher than that of the unmodified one. Sawhney et al.^3^ studied the performance of a SAH channel on absorber plate-modified structures having wavy-up delta form. The highest improvement in Nusselt number of 223% was observed than the flat plate with a forward pitch of 3 and 5 wave winglets at Re of 4000. Using bent delta-shaped vortex generators was tested by Baissi et al.^4^. They proposed correlations for Nu and f as a function of Re and roughness parameter. Saravanakumar et al.^5^ hypothesized that using fins and baffles improved roughened SAH. The effect of operational and geometrical variables is investigated using exergetic analysis. When the regulating parameters are optimized using the Genetic algorithm, the recommended SAH has a maximum exergy efficiency of 5.2%.

Khoshvaght-Aliabadi et al.^6^ showed that using twisted turbulators considerably affected the performance values, raising them by 2.21 and 7.79 times above the plane case. Heat transfer was improved by 4.52 when a V-shaped vortex generator was used. The fin height and pitch values of wavy fins were optimized by Haldar et al.^7^. A height of 0.7 mm and a pitch of 15 mm gave an extraordinary performance index of 1.96. Skullong et al.^8^ investigated the application of wavy grooves attached to winglets and discovered a performance index of 2.12 with a blockage ratio of 0.24. Arunkumar et al.^9^ demonstrated numerically that performance was found to be higher when the spring-shaped fins’ coil diameter decreased. Alam and Kim^10^ discovered that giving conical protrusion ribs significantly increased heat transmission compared to spherical ribs.

When a protrusion rib was used in SAH, Bhushan and Singh^11^ reported that the Nusselt number increased by 3.8 times and the friction factor by 2.2 compared to a plain duct. Bhattacharyya et al.^12^ conducted experiments in a circular-type heat exchanger using four angles of attack for inclined ribs. Kumar et al.^13^ investigated experimentally protrusion-shaped roughed SAH to improve the performance and discovered that the Nusselt number obtained is 108.23, about 245% greater than the smooth duct. Matheswaran et al.^14^ improved the performance of jet impingement SAH by using multiple arc protrusions as barriers, resulting in a 56.8% increase in exergetic efficiency. Gilani et al.^15^ used conical pin turbulators in the absorber plate of a SAH. It was determined that an absorber plate with a pin pitch of 16 mm may have an increased efficacy of 26.5%. Xiao et al.^16^ investigated the effect of inclined trapezoidal vortex generators (ITVGs) on heat transmission and flow behaviour in an SAH. According to the findings, the proposed SAH’s energy and exergy efficiency were around 24% and 31% higher than the comparable flat SAH.

The study by Kidane et al.^17^ analysed the energy and exergy efficiencies of two different dryers connected to a drying system and the drying kinetics of apple slices. The exergy inflow, outflow, and efficiency of the solar air heaters and drying chamber were evaluated. The results showed significant variations in energy and exergy efficiencies within the dryers and solar air heaters. The average efficiency of the dryers and solar air heaters was significantly higher on day 1 and day 2, respectively. The drying of apple slices occurred during the falling rate period. Ten thin-layer drying models were assessed for prediction. The investigations by Fan et al.^18^ discussed a system using a plate with V-shape ribs inside a tube as a turbulator to increase heat transfer rate. The system uses water-based hybrid nanofluids, including Al_2_O_3_–Cu/water, Cu–CuO/water, and Cu–TiO_2_/water. The study evaluates the effects of the type and volume concentration of the hybrid nanofluids on heat transfer enhancement. The results show that all hybrid nanofluids improved thermal performance compared to pure water. The differences are more significant for higher Reynolds numbers. The Cu–CuO/water hybrid nanofluid with a volume concentration of 1.5% has the highest thermal performance value.

Jovani et al.^19^ demonstrated that the use of extended surfaces or turbulators on absorber plates (AP) can significantly improve the thermal performance of solar air heaters (SAHs). However, there is a lack of parametric studies on the benefits of using coil springs as turbulators. An experimental analysis of AP equipped with vertical coil springs examined the effects of geometrical factors at three different levels. The research found that the heat transfer enhancement obtained using CSs is often greater than the resulting pressure drop, resulting in an overall performance index higher than unity. The highest Nusselt number and best overall performance were achieved at 6 mm. The overall performance of SAH declines with increasing coil pitch and diameter. A study by Jovini et al.^20^ analyzed the hydraulic and thermal characteristics of a solar air heater’s absorber plate with different springs and revealed that installing springs significantly impacts the heater’s performance. The effects of geometric parameter variations in the crossflow pattern were found to be higher than in the axial-flow pattern. The Nusselt number enhancements and friction factor augmentations were found to be in the range of 3.11–4.76 and 5.01–18.81 for the axial-flow pattern and 3.12–5.59 and 7.51–28.39 for the crossflow pattern.

The summary of the literature review on roughened absorber plates is depicted in Table 1.

**Table 1:** Literature showcasing artificial roughness on the absorber plate.

References	Types of fins used	Pictorial view of the finned model	Variables chosen	Major outcomes
Saravanan et al.^21^	Perforated C-shaped fin		Fin pitch = 1.6 to 1.9 cm Fin height = 1.5 to 3.5 cm Fin gap = 6 cm Diameter of fin = 8 cm Diameter of perforation = 1 cm Duct dimensions (length × width × height) = 143 cm × 70 cm × 5 cm Range of Re = 3000 to 24,000	The total heat transfer rate and the friction factor values increased by 2.67 and 5.34 times, respectively, compared with the plain duct
Sureandhar et al.^22^	Arc shaped rib type fins		Height ratio = 0.0422 and 0.0541 Pitch = 10 Angle of attack = 0.33 Dimensions of the duct = 0.3 m × 0.025 m Inlet flow rate = 1.2 to 3.6 kg/min	Variable arc rib fins outperformed fixed arc rib fins and plain ducts in heat transmission and thermo-hydraulic performance
Kumar et al.^23^	S-shaped ribs		e/D ranging 0.022–0.054, P/e ranging between 4 and 16, α fluctuating between 30° to 75°, W/w fluctuating between 1 and 4 Flow Re between 2400 and 20,000	The maximal augmentation in Nu has been estimated to correspond to e/D = 0.043, P/e = 8, α = 60°, W/w = 3 and the optimum value of THP = 3.34
Kumar et al.^24^	Twisted rib		Relative roughness pitch (P/e): 6 to 10 Rib orientation angle (α) 30° to 90° Twist ratio: 3 to 5	The configuration with pitch ratio = 8, twist ratio of 3 and orientation angle = 60° is deemed to provide the best results:
Yadav et al.^25^	Wire rib		Rib pitch variation from 10 to 25 mm Reynolds number varied from 3800 to 18,000 Correlations are developed for Nu and f	Developed correlations predict f and Nu lie in the ± 5% deviation line
Navneet Arya et al.^26^	Dimpled roughness		The angle of attack (α) varied from 45 to 75◦ Relative long way (RLL) length (l/d) varied between 15 and 25 Relative height (roughness) variation is in between 0.024 and 0.036 Relative wire length is ranging between 0.14 and 0.21 Reynold’s number ranges from 5000 to 20,000	THP reached a maximum value of 1.63 for the parameters α = 45°, l/d = 20, and w/D_h_ = 0.18
Subhash Chand et al.^27^	Louvered fins		The mass flow rate is ranged between 0.0070 and 0.0158 kg/s angle = 20° length = 25 mm, pitch = 25 mm Height = 35 mm Fin spacing is varied from 2 to 5 cm	The thermal efficiency of the louvred fin is found to be 106.7% higher than the base model for the fin spacing of 2 cm
Misra et al.^28^	V-down ribs with multiple gaps and turbulence promoter		The pitch is varied between 8 and 14 in steps of 2 The attack angle increased from 45° to 60° in step 5° Hydraulic Diameter = 0.03 m Relative Roughness Height = 0.04	Maximum performance was achieved at P/e = 10 and angle of attack 45°, resulting in 2.26 times increase in Nu
Patel et al.^29^	Usage of reverse NACA profile ribs		Reynolds number is varied from 6000 to 18,000 Spacing between two ribs = 25 mm relative roughness height varied from 0.043 to 0.065	At Re = 18,000, a maximum Nu of 104.45 was obtained The optimum THEF of 2.53 was obtained with Re = 6000
Patel S et al.^30^	V-ribs with symmetrical gaps and staggered ribs		Angle of attack = 60° Number of gaps ranging from 1 to 4 in steps of 1 The ratio of staggered rib position to pitch = 0.65 Re fluctuated from 4000 to 14,000	The maximum Nu and f augmentations were 2.05 and 3.39 times, respectively

Considering the literature mentioned above review, various researchers have dedicated significant time and effort to creating SAHs with effective absorber designs to increase their performance. Regardless of the large number of works on SAH, there have been a few works evaluating their effectiveness from an energy and exergy aspect, particularly for freshly erected solar air heaters. Furthermore, based on the energy and exergy studies, additional research is needed to evaluate the effectiveness of solar air heaters when using capsule-shaped turbulators. As a result, the fundamental purpose of this work is to numerically investigate the performance of an SAH fitted with a newly developed absorber plate equipped with capsule-shaped turbulators. The enlarged surface area of the SAH absorber and the smooth splitting of flow at the turbulator’s end surface are the primary advantages of this design over previous designs. The performance of this new design is assessed utilizing energy and exergy methodologies. The investigation is carried out on a single pass airflow with a Reynolds number ranging from 3000 to 21,000.

Using capsule-shaped turbulators in solar air heaters introduces a novel approach to enhancing heat transfer by creating complex flow patterns that improve turbulence intensity. Unlike conventional turbulators, the capsule shape promotes swirl and secondary flows, leading to better thermal performance with minimal pressure drop. This design optimizes the heat transfer coefficient by increasing the contact between heated surfaces and the working fluid, thereby improving overall efficiency. The streamlined nature of the capsule-shaped elements reduces flow resistance compared to sharp-edged turbulators, balancing thermal enhancement with aerodynamic efficiency. By integrating capsule-shaped turbulators, solar air heaters can achieve higher thermal efficiency, making them more effective for renewable energy applications.

Furthermore, the performance of the developed solar air heater is compared to that of the standard flat solar air heater under all flow circumstances. This has an immediate influence on SDG 7, one of the 17 SDGs set forth by the UN for the year 2030, and contributes to increasing the share of renewable energy in the global energy situation. This complete analysis is vital for ensuring optimal performance while remaining cost-effective and environmentally friendly.

## Performance parameters

### Nusselt Number

Nusselt number of the flow is calculated using Eq. (1) as

1 $$N_{u} = \frac{{h_{c} L_{c} }}{k}$$

### Friction factor

Darcy Weisbach equation is applied to compute the friction factor for the solar air heater across the test segment and is given by Eq. (2)

2 $$f = \frac{{\Delta pL_{c} }}{{2\rho LV^{2} }}$$

### Absorber plate temperature index

It is computed by Eq. (3) as

3 $$C_{ap} = \frac{{T_{top\_s} - T_{1} }}{{T_{top\_t} - T_{1} }}$$

### Thermal enhancement factor (TEF)

Webb and Eckert^31^ proposed a thermal enhancement factor that calculates the air heater’s thermal and hydraulic performance. Here, a comparison between a roughened and smooth duct (without turbulators) is presented. TEF is given by Eq. (4)

4 $$TEF = \frac{{\left( {N_{u\_t} /N_{u\_s} } \right)}}{{\left( {f_{t} /f_{s} } \right)^{1/3} }}$$

### Thermo-hydraulic performance parameter (THPP)

Lewis (1975) developed an additional parameter called the Thermo-hydraulic performance parameter, which is given by Eq. (5). The THPP typically combines the thermal and hydraulic performance of a heat exchange system, often by factoring in heat transfer effectiveness and pressure drop or flow resistance. When the Stanton number is used, it provides insight into the heat transfer characteristics, which can be incorporated into the THPP.

5 $$THPP = \frac{{\left( {S_{t\_t} /S_{t\_s} } \right)^{3} }}{{\left( {f_{t} /f_{s} } \right)}}$$

### Exergy efficiency

Exergy analysis, which depends on the second rule of thermodynamics, is more rational than energy analysis because it focuses on a thermal system’s roots, locations, and inefficiencies. Exergy is a feature of system-environment combinations that embodies the maximum amount of work that can be given without breaching the rules of thermodynamics. It is quantified with the help of exergy efficiency and is given as Eq. (6)^32^.

6 $$\eta_{II} = \frac{{Ex_{2} }}{{Ex_{1} }} = \frac{{\dot{m}\left( {\Delta h - T_{e} \Delta s} \right)}}{{\left( {1 - T_{amb} /T_{top} } \right)Q_{s} }}$$

The exergy efficiency evaluates how effectively the system converts solar radiation into useful work or exergy. Since exergy accounts for irreversibility, such as heat losses and entropy generation, it provides a better measure of system performance than first law (thermal) efficiency.

### The Sustainability Index (SI)

This is a novel indicator for assessing process inefficiency, production system inefficiency, and the consequences. These three parameters are based on an exergy analysis that ensures the process’s irreversible energy and sustainability^33^. Equation (7) gives the mathematical formula for the Sustainability Index.

7 $$SI = \frac{1}{{1 - \eta_{II} }}$$

## Numerical formulation

### Computational geometry

**Fig. 1:**
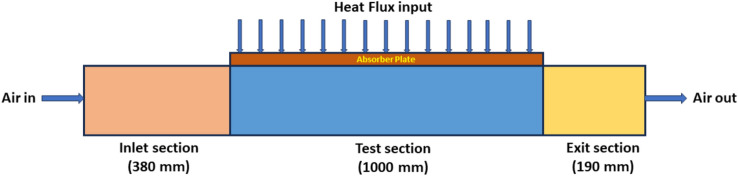
Geometric details of SAH.

The various dimensions of the SAH used in this investigation are depicted in Fig. 1. The solar air heater is numerically analyzed and divided into three sections: the entrance duct, the exit duct, and the test portion. The heater’s inlet and outlet duct lengths are modelled according to the ASHRAE standard 93–97^34^, which prescribes the most petite length along the inlet and the exit as $5\sqrt {WH}$ and $2.5\sqrt {WH}$ respectively. The length of the entrance duct is 380 mm, while the output duct is 190 mm. The input duct measures 200 mm in diameter by 30 mm high.

**Fig. 2:**
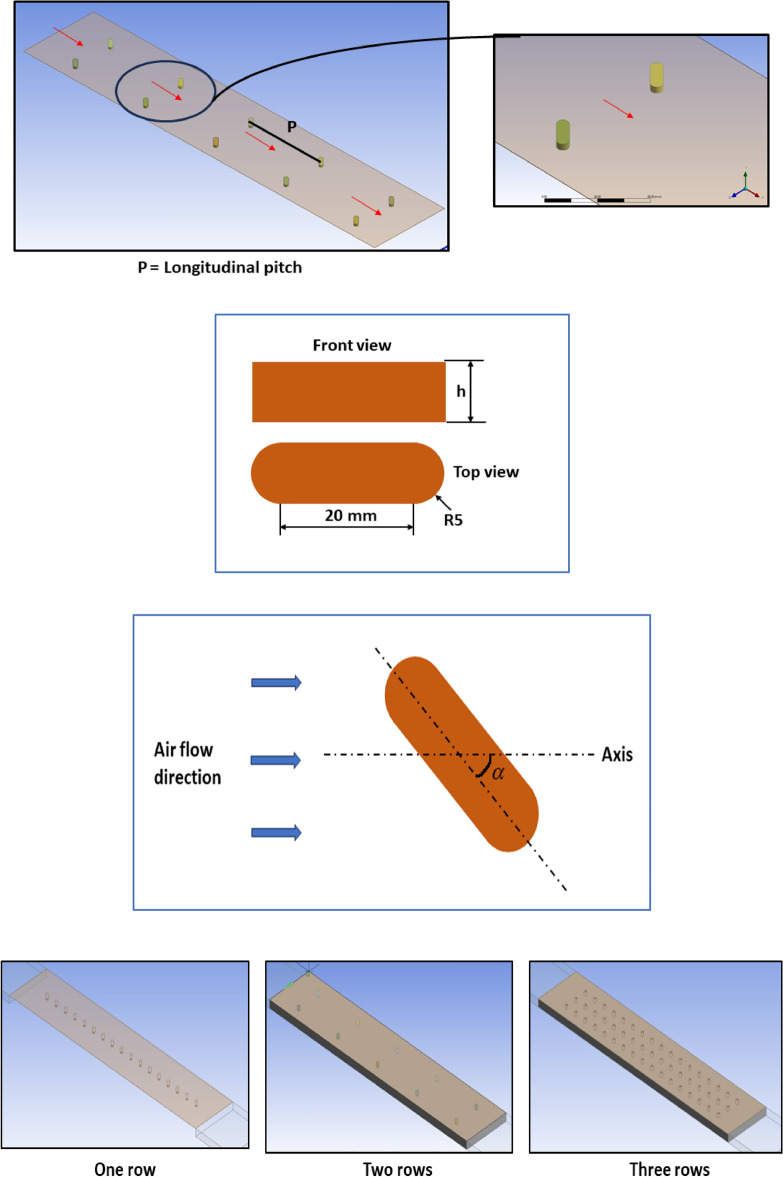
Capsule-shaped turbulators used in the analysis.

The capsule-shaped turbulators (considered to be made of copper) are inserted underneath the absorber duct or plate. Figure 2 depicts the turbulator structure and dimensions.

The length (in the flow direction) and width (perpendicular to the flow direction) of the turbulator are maintained constant at 20 mm and 10 mm, respectively. The side curvature of the capsule shape is built with a constant arc radius of 5 mm. The alignment of the turbulator is defined in terms of the orientation angle ($\alpha$), measured as the angle between the centreline of the turbulator and the flow direction. This orientation angle varies from 0° to 90° in steps of 15°. The longitudinal pitch ratio (*P*/*L*) varies from 0.025 to 0.25 with an increment of 0.05. The height ratio (*h*/*L*) of the turbulator varies from 0.004, 0.005, 0.006, 0.007, and 0.008. Turbulator rows are adjusted from 1 to 3 for each arrangement with uniform spacing. Variations in the geometrical parameters of the turbulator configuration are depicted in Table 2.

**Table 2:** Geometrical parameters of the turbulators chosen for the study.

Parameters	Dimensional variation	Non-dimensional variation
Orientation Angle	0°, 15°, 30°, 45°, 60°, 75°, 90°	–
Longitudinal Pitch	25, 50, 100, 150, 200, 250	0.025, 0.05, 0.1, 0.15, 0.2, 0.25
Turbulator Height	4, 5, 6, 7, 8	0.004, 0.005, 0.006, 0.007, 0.008
No. of rows	One, Two, Three	–

A lower pitch ratio (e.g., 0.025) results in strong recirculation zones and enhances heat transfer, possibly leading to a higher pressure drop. A higher pitch ratio (e.g., 0.25) allows for better flow uniformity and reduced pressure drop, but the heat transfer enhancement may decrease. The selected range is chosen to balance heat transfer enhancement and pressure drop, ensuring that there must be a trade-off condition.

### Equations governing the numerical scheme

The fluid flow equations, based on the continuity and Navier–Stokes equations, describe air motion in the duct, with the momentum equation incorporating convective acceleration, pressure gradients, and viscous effects.

The equations guiding the numerical scheme are as follows^35^:

8 $$\frac{{\partial \overline{u} }}{\partial x} + \frac{{\partial \overline{v} }}{\partial y} + \frac{{\partial \overline{w} }}{\partial z} = 0\quad {\text{for}}\,{\text{continuity}}$$

9 $$\frac{\partial }{{\partial x_{i} }}(\rho \overline{{u_{i} }} \overline{u} ) = \frac{{\partial \overline{p} }}{{\partial x_{i} }} + \frac{\partial }{{\partial x_{i} }}\left( {\mu \left( {\frac{{\partial \overline{{u_{i} }} }}{{\partial \overline{{x_{j} }} }} + \frac{{\partial \overline{{u_{j} }} }}{{\partial \overline{{x_{i} }} }} - \frac{2}{3}\delta_{ij} \frac{{\partial \overline{{u_{l} }} }}{{\partial x_{l} }}} \right)} \right) - B_{i} - \frac{\partial }{{\partial x_{i} }}\left( {\overline{{\rho u^{\prime }_{i} u^{\prime }_{j} }} } \right)\quad {\text{for}}\,{\text{momentum}}$$

Reynolds stress is estimated using the Boussinesq hypothesis as follows:

10 $$- \rho \left( {\overline{{u_{i}^{\prime } u^{\prime }_{j} }} } \right) = \mu_{t} \left( {\frac{{\partial u_{i} }}{{\partial x_{j} }} + \frac{{\partial u_{j} }}{{\partial x_{i} }}} \right) - \frac{2}{3}\left( {\rho k + \mu_{t} \frac{{\partial u_{k} }}{{\partial x_{k} }}} \right)\delta_{ij}$$

#### Energy equation

The energy equation for a solar air heater ensures the conservation of heat energy by balancing the absorbed solar radiation, convective heat transfer to the air, radiative exchange with the glass cover, and heat losses to the surroundings^35^.

11 $$\rho \frac{\partial h}{{\partial t}} = K_{s} \left( {\frac{{\partial^{2} T}}{{\partial n^{2} }}} \right) + S_{h}$$

where

12 $$e_{t} = \left( {h - \frac{p}{\rho } + \frac{{V^{2} }}{2}} \right) = {\text{specific}}\,{\text{energy}}.$$

The energy of a solid expanse, such as an absorber plate or other wall region, can be calculated as follows:

13 $$\rho \frac{\partial h}{{\partial t}} = K_{s} \left( {\frac{{\partial^{2} T}}{{\partial n^{2} }}} \right) + S_{h}$$

#### Re-Normalisation Group (RNG) k-ε model

The Re-Normalisation Group (RNG) k-ε model is an advanced turbulence model used in the numerical simulation of a solar air heater to improve accuracy in predicting heat transfer and airflow characteristics. It refines the standard k − $\varepsilon$ model by incorporating additional terms in the transport equations, allowing better modelling of turbulence dissipation and flow separation effects.

14 $$\frac{\partial (\rho k)}{{\partial t}} + \frac{{\partial (\rho ku_{i} )}}{{\partial x_{i} }} = \frac{\partial }{{\partial x_{j} }}\left[ {\frac{{\mu_{t} }}{{\sigma_{k} }}\,\,\frac{\partial k}{{\partial x_{j} }}} \right] + 2\mu_{t} E_{ij} E_{ji} - \rho \varepsilon$$

15 $$\frac{\partial (\rho \varepsilon )}{{\partial_{t} }} + \frac{{\partial (\rho \varepsilon u_{{_{i} }} )}}{{\partial x_{i} }} = \frac{\partial }{{\partial x_{j} }}\left[ {\frac{{\mu_{t} }}{{\sigma_{\varepsilon } }}\,\,\frac{\partial \varepsilon }{{\partial x_{j} }}} \right] + C_{1\varepsilon } \frac{\varepsilon }{k}2\mu_{t} E_{ij} E_{ji} - C_{2\varepsilon } \rho \frac{{\varepsilon^{2} }}{k}$$

The constants from Eqs. (14) and (15) are given below^35^:

$$C_{\mu } = 0.09;\sigma_{k} = 1.00;\sigma_{\varepsilon } = 1.30;C_{1\varepsilon } = 1.44;C_{2\varepsilon } = 1.92.$$

### Boundary conditions

- At the inlet duct, the flow Reynolds number varies between 3000 and 21,000 in steps of 3000, corresponding to the range of mass flow rates between 0.006 and 0.04 kg/s.
- Considering the pressure outlet condition, a pressure value of 1.01325 bar is fixed at the exit duct.
- A steady heat flux of 900 W/m^2^ is applied to the absorber plate’s top surface. This heat flow model represents the average solar heat flux experienced by a solar air heater.
- Surrounding air temperature of 298 K is set in the operating conditions.
- The gravity effect is negligible.

### FLUENT solver settings

The solver settings listed below are used to carry out the numerical analysis.

- The fluid flow is considered to be turbulent, constant and fully developed.
- Second-order upwind techniques solve the spatial discretization for momentum, energy, and continuity to produce an accurate answer.
- Convergence occurs when the residuals for energy and momentum fall below 10^–8^ and 10^–6^, respectively.
- The walls have no-slip boundary conditions.

### Characteristic properties of air

Air-related properties, such as air density, air viscosity, and thermal conductivity, are temperature-dependent, as shown by the following equations^36^:

16 $$\rho = 0.01\left( {391.47\,\, - \,\,1.6082\,\,T\,\,\, + \,\,\,290.13 \times 10^{ - 5} \,T^{2} \,\,\, - \,\,\,194.07 \times 10^{ - 8} } \right)\, \times T^{3}$$

17 $$\mu \,\, = \,\,\left( {161.57\,\, + \,\,6.523T\,\, - \,\,302.97\,\, \times \,\,10^{ - 5} \,\,T^{2} \,\,} \right)\,\, \times \,\,10^{ - 8}$$

18 $$K\,\, = \,\,(0.15215\,\, + \,\,9.746\,\,T\,\, - \,\,333.22\,\, \times \,\,10^{ - 5} \,\,T^{2} \,) \times 10^{ - 5}$$

### Meshing and Mesh sensitivity analysis

**Fig. 3:**
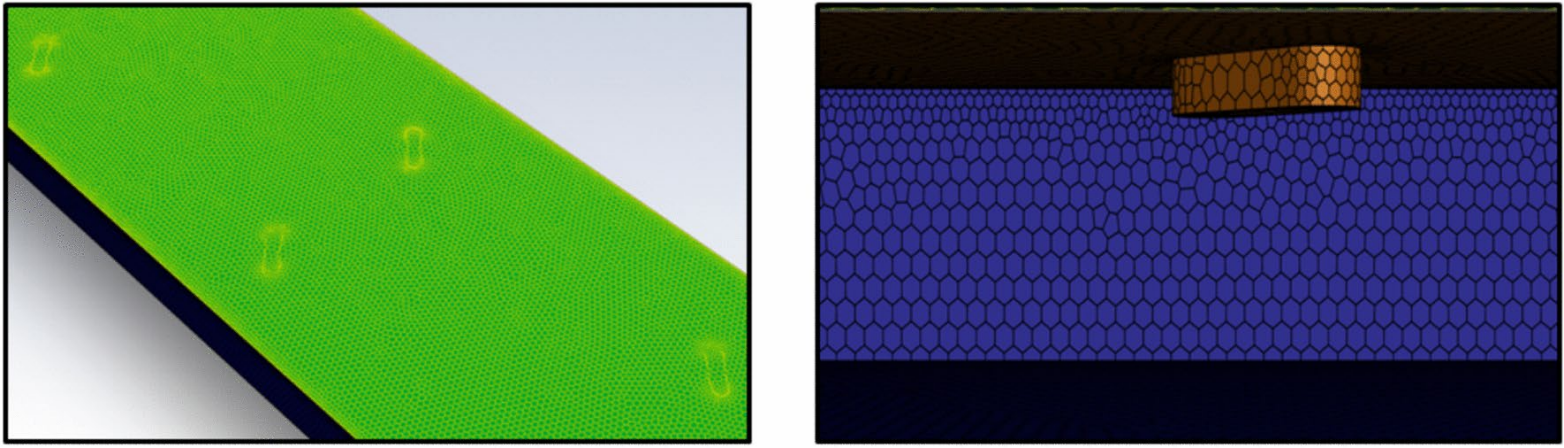
Localized view of meshed domain.

The mesh is created using the Ansys Workbench. Boundary layer meshes are inserted at the fluid side of the absorbing duct to capture the boundary layer effect. Figure 3 depicts the meshed domain localized view with turbulators. The absorber plate is discretized using poly-hexacore mesh.

A mesh sensitivity examination was undertaken to ensure the results were independent of the mesh size. Four models with varied mesh densities are investigated, and the study is carried out for a mass flow rate of 0.006335 kg/s, corresponding to Re = 3000.

**Fig. 4:**
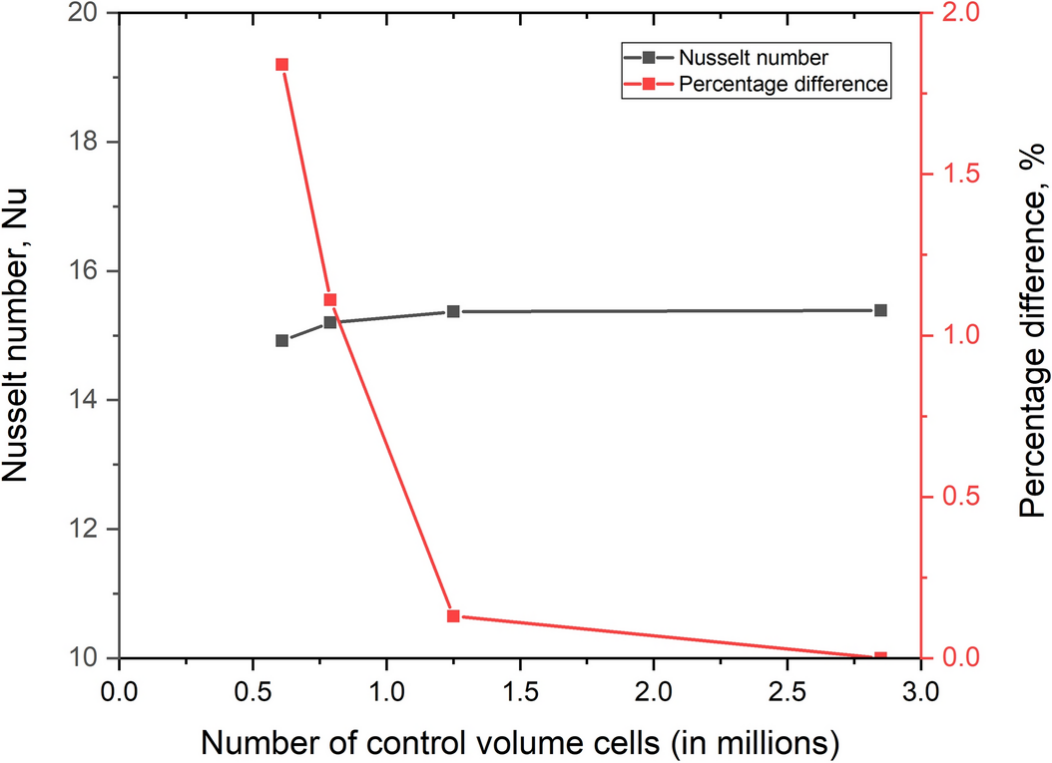
Mesh sensitivity analysis.

Table 3 and Fig. 4 illustrate the Nusselt number for mesh densities of 0.61, 0.79, 1.25, and 2.85 million elements, respectively. It is observed that when the number of elements increased from 1.25 million to 2.85, Nu varied by 0.13%. The investigation used 1.25 million elements, considering calculation time and costs.

**Table 3:** Results of Mesh sensitivity analysis.

Number of elements (in millions)	Nusselt number	Percentage difference
2.85	15.39	–
1.25	15.37	0.13
0.79	15.20	1.11
0.61	14.92	1.84

To accurately capture the heat transfer from the absorber surfaces to the working fluid, the mesh is refined close to the wall surface to yield the y+ values near unity and is equal to 1.04. The meshing is done using a poly-hexacore meshing module of Ansys Fluent, the new technique for achieving the desired y+ values and thereby ensuring the finer meshing near the wall boundaries with gradual increase in mesh size towards the central stream.

### Selection of turbulence model

Table 4 lists several models that have been utilized in the literature. Out of the different available models, the RNG k–ε turbulence model was chosen above the other available models in this study because of its higher accuracy.

**Table 4:** Turbulence Models used in literature.

Sl No	References	Turbulator design	Chosen turbulence model
1	Bensaci et al.^37^	SAH with various baffle positions	RNG k-ε
2	Khanlari et al.^38^	Solar collector with shaped perforated baffles	RNG k-ε
3	Bezbaruah et al.^39^	SAH with half-conical truncated vortex generators	RNG k-ε
4	Arunkumar et al.^36^	SAH with rectangular duct with perforations	RNG k-ε
5	Xiao et al.^16^	Inverted trapezoidal vortex generators for solar air heaters	RNG k-ε
6	Present analysis	Solar air heater with capsule-shaped turbulator	RNG k-ε

### Validation studies

Before starting the numerical work, validating the CFD model with established correlations available in the literature for the baseline model of solar air heaters (without turbulators) is necessary. Predicted results for Nu with Re ranging from 3000 to 21,000 are compared with those obtained from Gnielinski’s correlation (Eq. 19) and Dittus Boelter equation (Eq. 20).

19 $$Nu = \frac{{\left( {f/8} \right) \times \left( {{\text{Re}} - 10^{3} } \right) \times \Pr }}{{1 + \left[ {12.7 \times \sqrt {f/8} \times \left( {\Pr^{2/3} - 1} \right)} \right]}}$$

20 $$Nu = 0.023{\text{Re}}^{0.8} \Pr^{0.4}$$

**Fig. 5:**
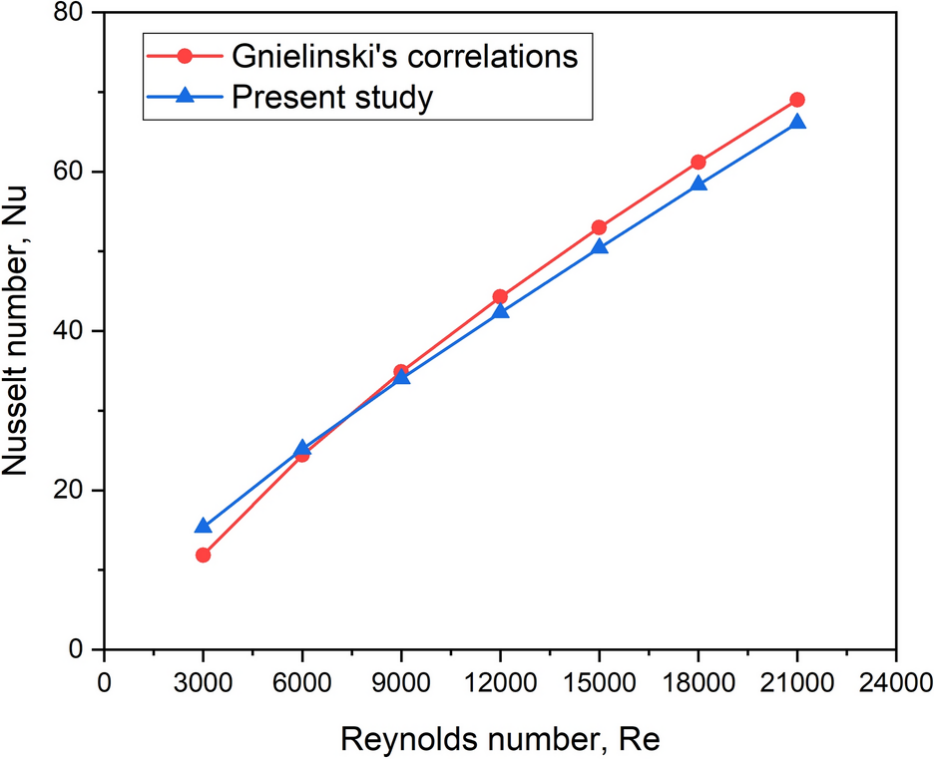
Validation study for Nu.

Figure 5 compares the numerically obtained Nu and the Nu predicted by Eq. (19). The largest deviation of the numerically derived Nu from Gnielinski’s correlations was 7.1%.

Similarly, the numerically computed friction factor (f) was calculated and compared to the Blasius equation^40^.

The Blasius equation is written as follows:

21 $$f = 0.391 \times {\text{Re}}^{ - 0.25}$$

**Fig. 6:**
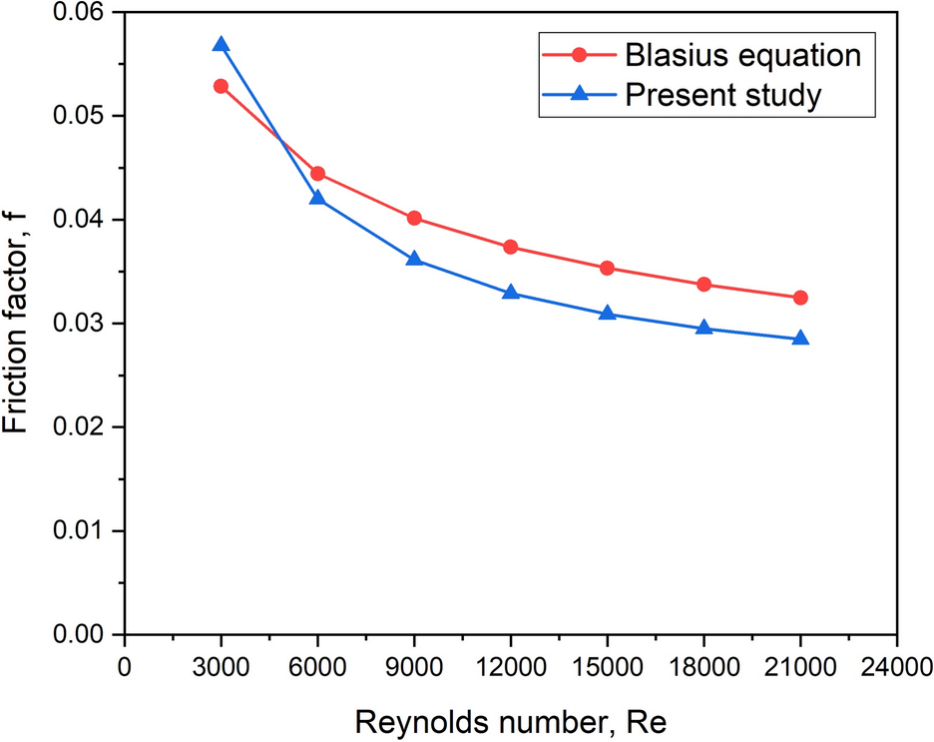
Validation study for f.

Figure 6 represents the variation of the friction factor obtained theoretically (using Eq. (21)) and numerically. The maximum deviation in the value of the friction factor between them was found to be ± 6.8%.

The minor deviations seen in these values are due to the truncational and rounding-off errors. As a result, the numerical model utilized in the research is clearly in good accord with the known relationships. Hence, the CFD model is assumed to be reliable in predicting reasonably acceptable results in terms of heat transfer and pumping characteristics.

**Fig. 7:**
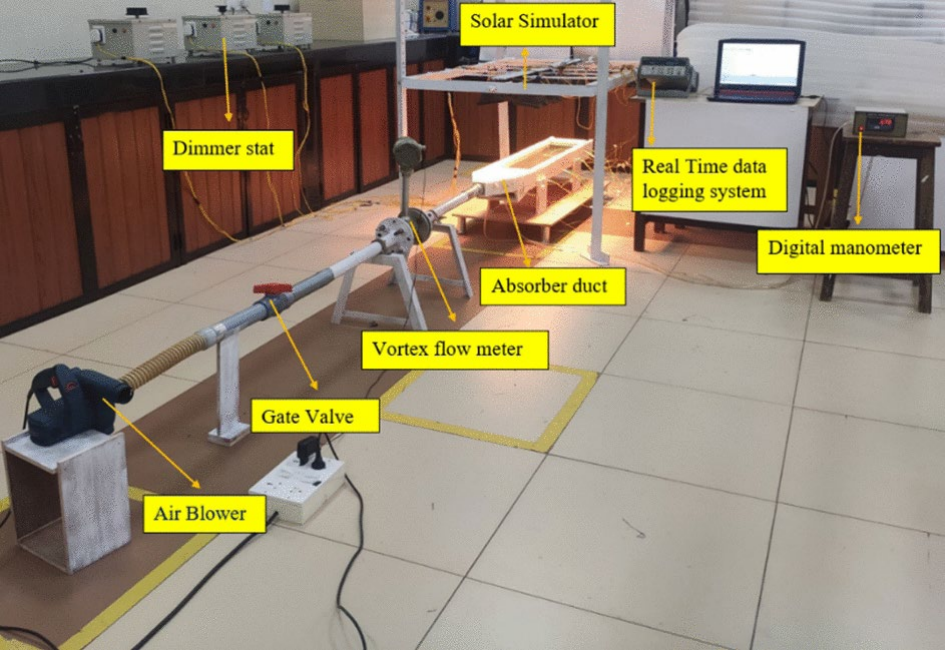
Experimental setup used for the validation studies.

Besides analytical validation, experimental validation is performed to affirm the results obtained. Figure 7 depicts the experimental configuration of the solar air heater used in this investigation. The extruded polystyrene foam insulation is placed on the exterior of the duct, and plywood is used to construct the experimental test rig. Black mat finish paint is applied to the absorber plate fixed to the absorber duct. Shiny laminates are installed on the inside surface of the duct to minimize flow losses caused by skin friction. The collector’s outlet is linked to a vortex meter to measure the air volume flow rate and a gate valve to control flow. Attaching the collector to a dual blower arrangement at the downstream end creates airflow inside the duct. Twelve "K" Type thermocouples are attached to the plate at designated locations to measure the absorber plate’s average temperature. Using an RTD-type thermocouple, the incoming and outgoing air temperatures are measured.

The pressure drop caused by flow resistance across the duct is measured with a digital manometer. A solar simulator is used to simulate the heat flux from solar insolation. Twelve halogen bulbs comprise the solar simulator’s array, and three dimmer stats adjust the simulator’s intensity.

**Fig. 8:**
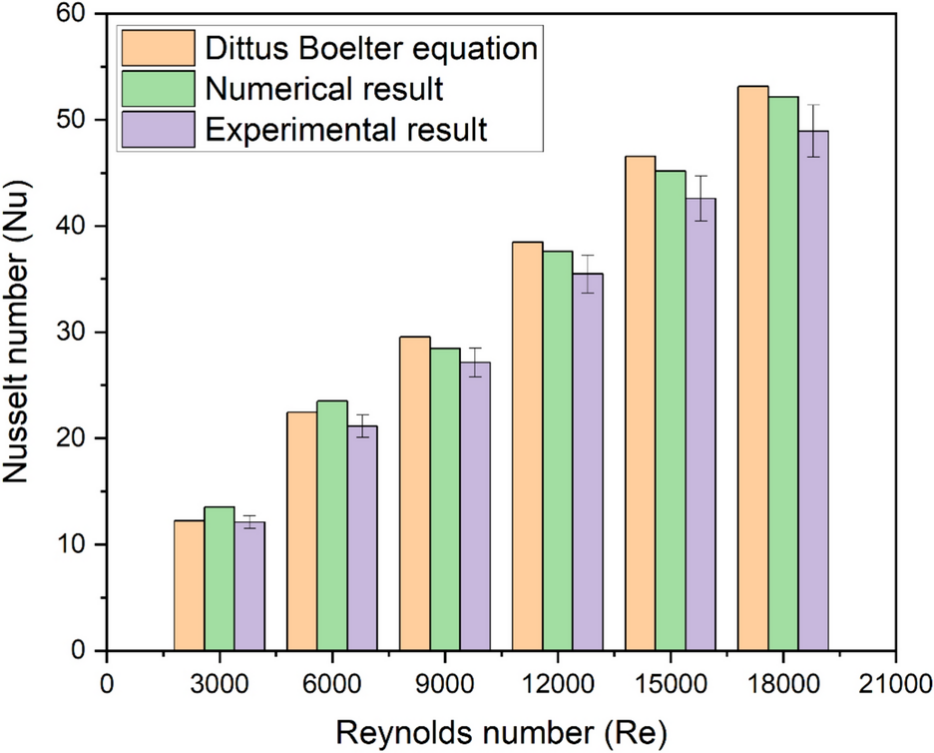
Validation plot for Nu.

Experimental results validate the Nu derived from numerical simulations. The Dittus Boelter Equation (using Eq. (19)) and the numerical results of the Nu are presented in Fig. 8, where they are compared with the experimental data, which agree quite well. The experimental results and the CFD anticipated outcomes are within a 10% range.

**Fig. 9:**
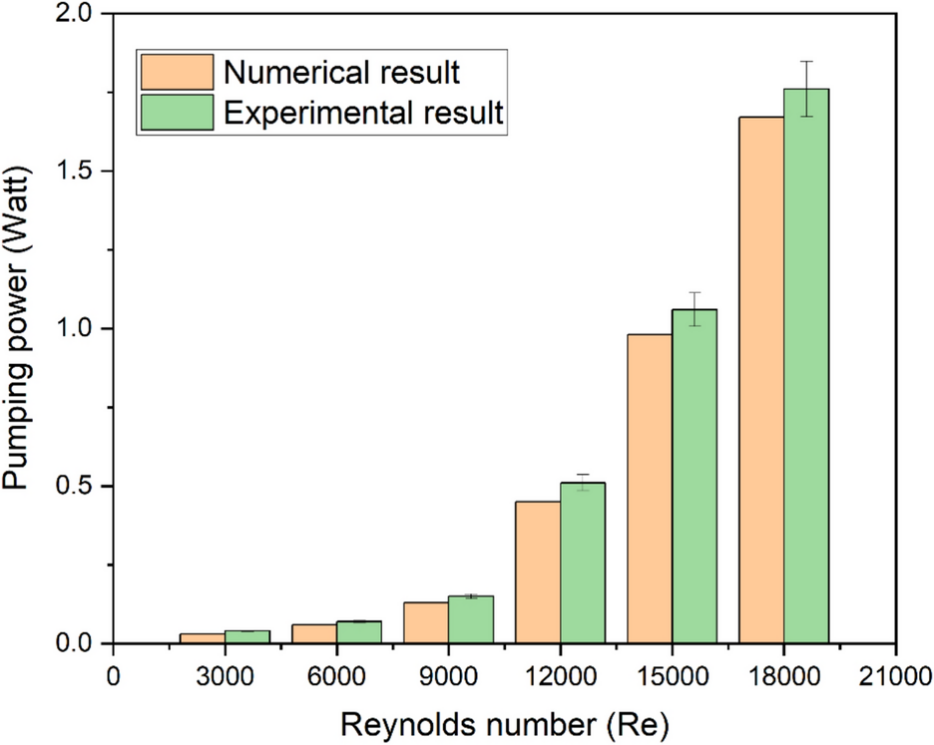
Validation plot for f.

Plotting the comparison results between the experimental and estimated pumping power requirements is done, as illustrated in Fig. 9. Observations reveal a 10% variation between the experimental and CFD projected findings.

The estimation of uncertainty in the calculated result ‘R’ is given by Eq. (22)^36^:

22 $$w_{R} = \left[ {\left( {\frac{\partial R}{{\partial x_{1} }}w_{1} } \right)^{2} + \left( {\frac{\partial R}{{\partial x_{1} }}w_{1} } \right)^{2} + \cdots + \left( {\frac{\partial R}{{\partial x_{n} }}w_{n} } \right)^{2} } \right]^{1/2}$$

where $R = f(x_{1} ,x_{2} ...x_{n} )$ and w_R_ is the uncertainty in the measurement of R. The uncertainties involved in the measurement of independent variables such as radiation heat flux, temperature, volume flow rate and absorber plate surface area are listed in Table 5.

**Table 5:** Uncertainties in the measurement of independent variables.

Measured variable	Uncertainty level
Volume flow rate	± 0.0001 m^3^/h
Temperature	± 0.001 K
Radiation heat flux	± 1 W/m^2^
Absorber plate length	± 1 mm
Absorber plate width	± 1 mm

Using Eq. (22), for the lower to higher Re range, the uncertainty in the Nusselt number is 1.2% to 3.6%, and the pumping power is 1.3% to 2.8%, respectively.

## Results and discussion

**Fig. 10:**
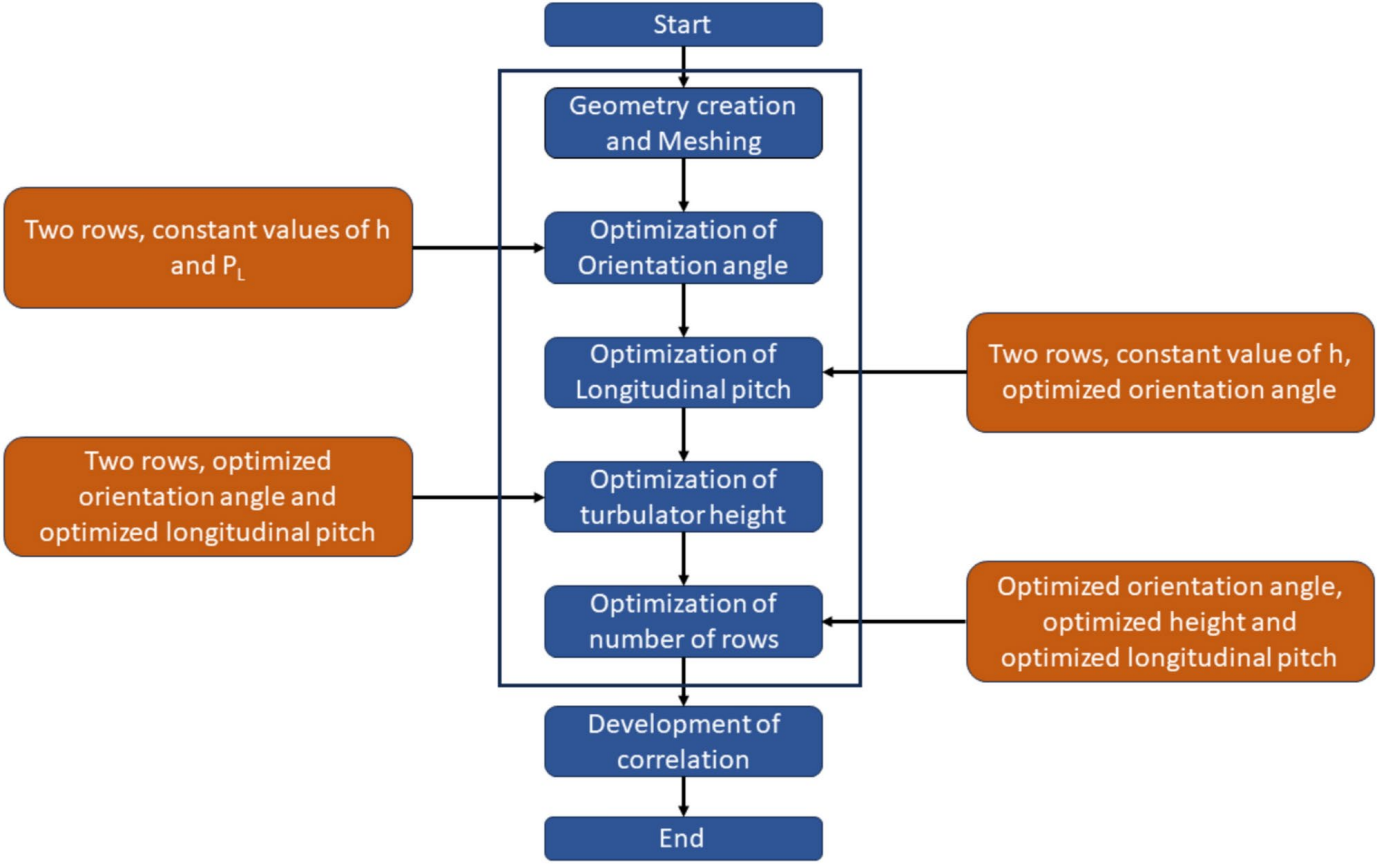
Structure of CFD analyses.

This section describes the performance characteristics of an air heater fitted with capsule-shaped turbulators. The findings are graphed to show the consequence of turbulators on the energy exchange behaviour SAH as compared with the base model, i.e., absorber plate without turbulators—the structure of the numerical analyses depicted in Fig. 10.

### Variation of orientation angle

A two-row turbulator arrangement is employed with a longitudinal pitch ratio of 0.2 and a turbulator height ratio of 0.005. The orientation angle varies from 0° to 90° in steps of 15°. The results obtained are discussed below.

#### Effect on Nusselt number

**Fig. 11:**
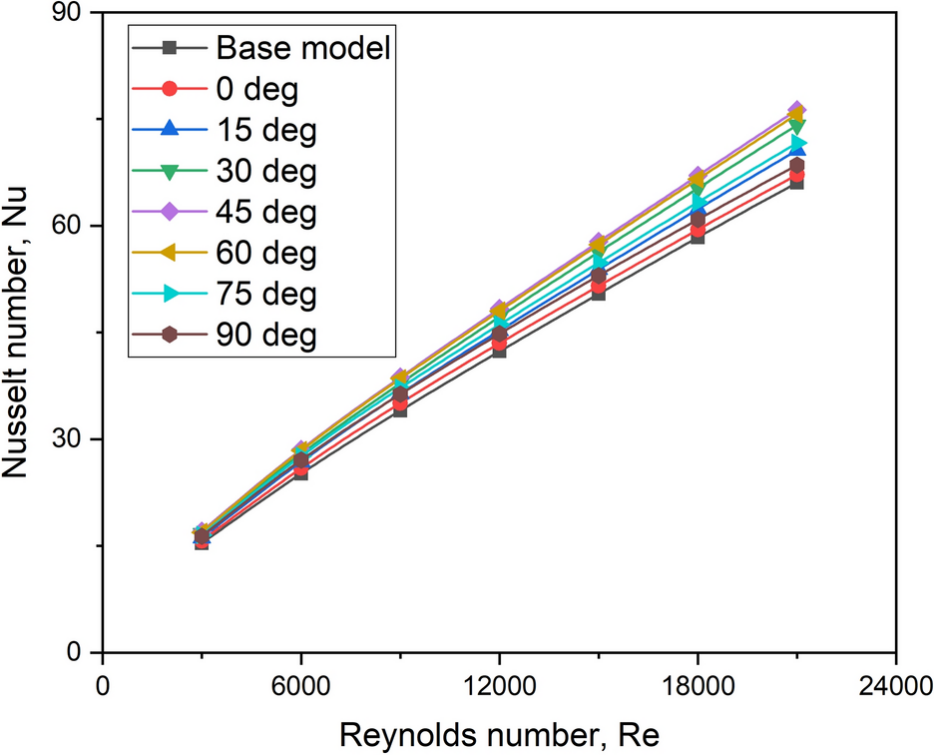
Nu versus Re for all orientation angles.

Figure 11 depicts the fluctuation of Nu with Re for various turbulator orientation angle values. For all turbulator orientation angle configurations, it is evident that upsurging the Reynolds number raises the Nusselt number. This is because an increase in Reynolds number tends to increase the severity of turbulence, which raises the heat transfer rate of the moving fluid. As is well-established, an increase in Re primarily increases fluid velocity and generates greater mixing inside the turbulent boundary layer, hence hastening the increase in Nusselt number. Higher Re increases the laminar sub-layer, resulting in better heat transfer due to the presence of eddies and circulations induced by the turbulators.

**Fig. 12:**
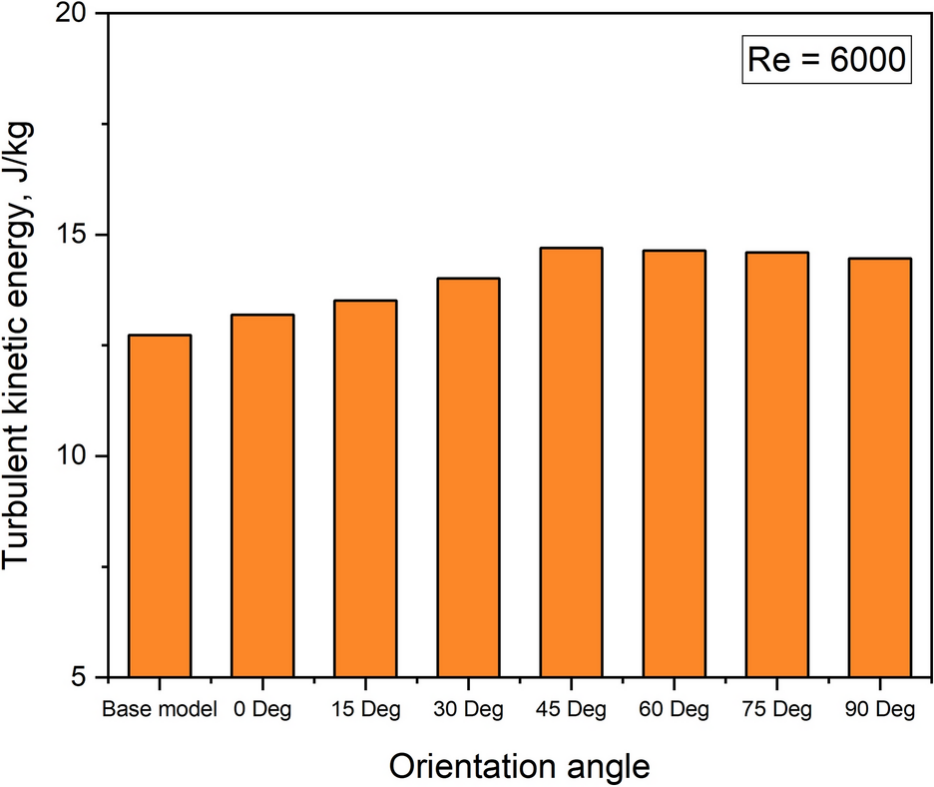
Turbulent kinetic energy values for various orientation angles.

Figure 11 further shows that, for all of the Reynolds values studied, the Nusselt number increases as the turbulator orientation angle increases from 0° to 45°, reaches an optimum value at 45° and then declines for the orientation angles from 45° to 90°. This is because up to the optimum value of orientation angle, the turbulator splits the flow evenly in the vicinity of curved regions of the turbulator on both sides, thereby increasing the turbulent kinetic energy as seen in Fig. 12. Beyond the optimum value, the turbulator acts like an obstruction and turbulent kinetic energy decreases thereby contributing towards a decremental trend for Nusselt number.

**Fig. 13:**
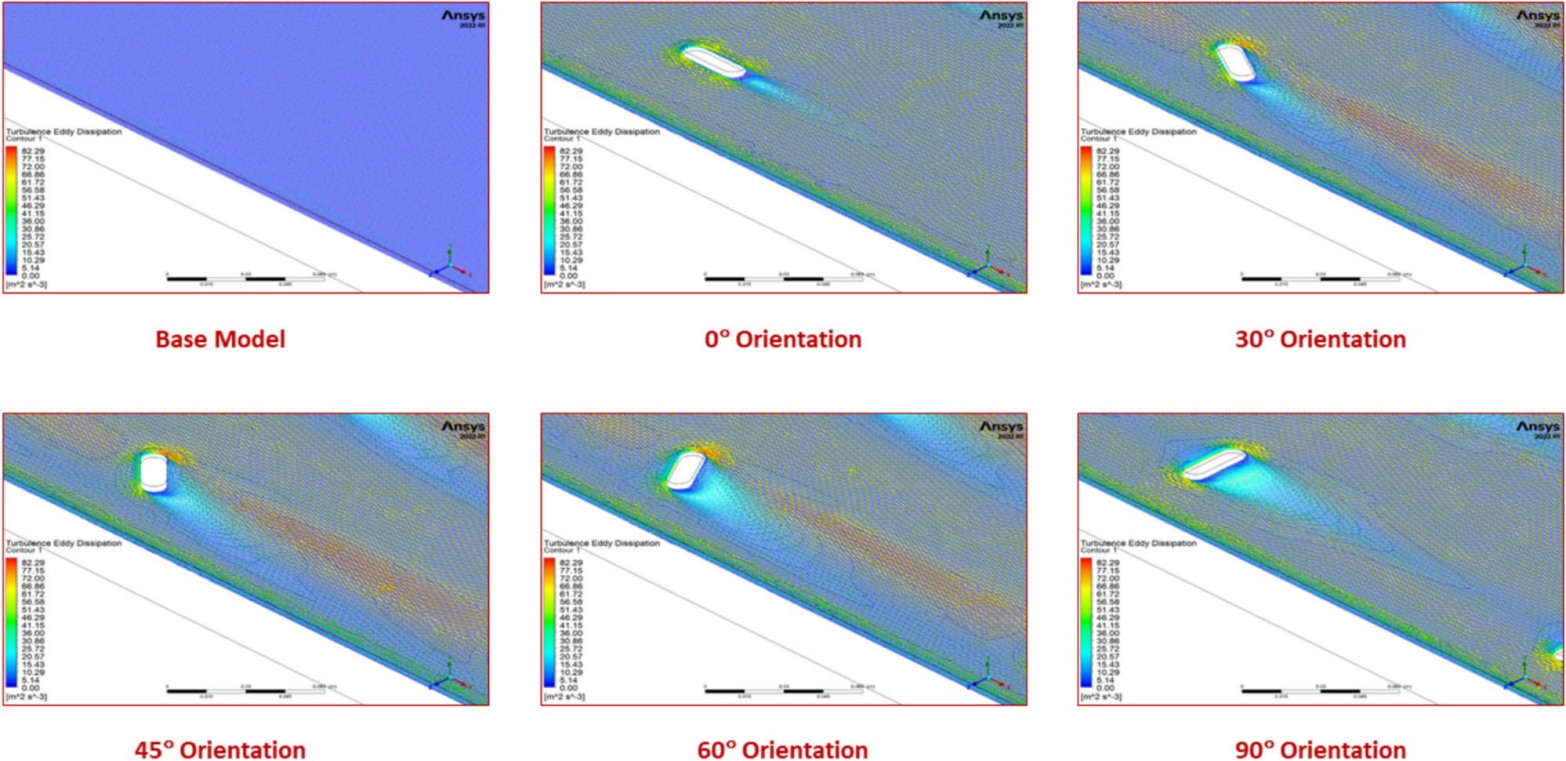
Contour plots of turbulent kinetic energy for various orientation angles.

This phenomenon is corroborated with the help of contour plots of turbulent kinetic energy, as seen in Fig. 13.

#### Effect on friction factor

**Fig. 14:**
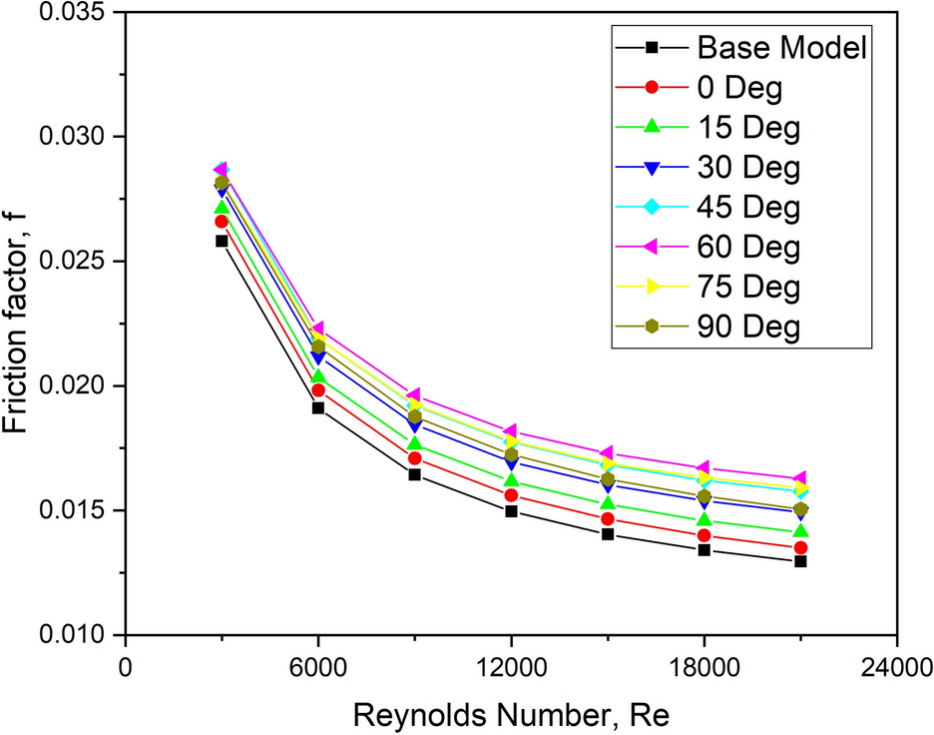
f versus Re for various orientation angles.

Figure 14 shows the fluctuation of friction factor with Re for various turbulator orientation angle values.

**Fig. 15:**
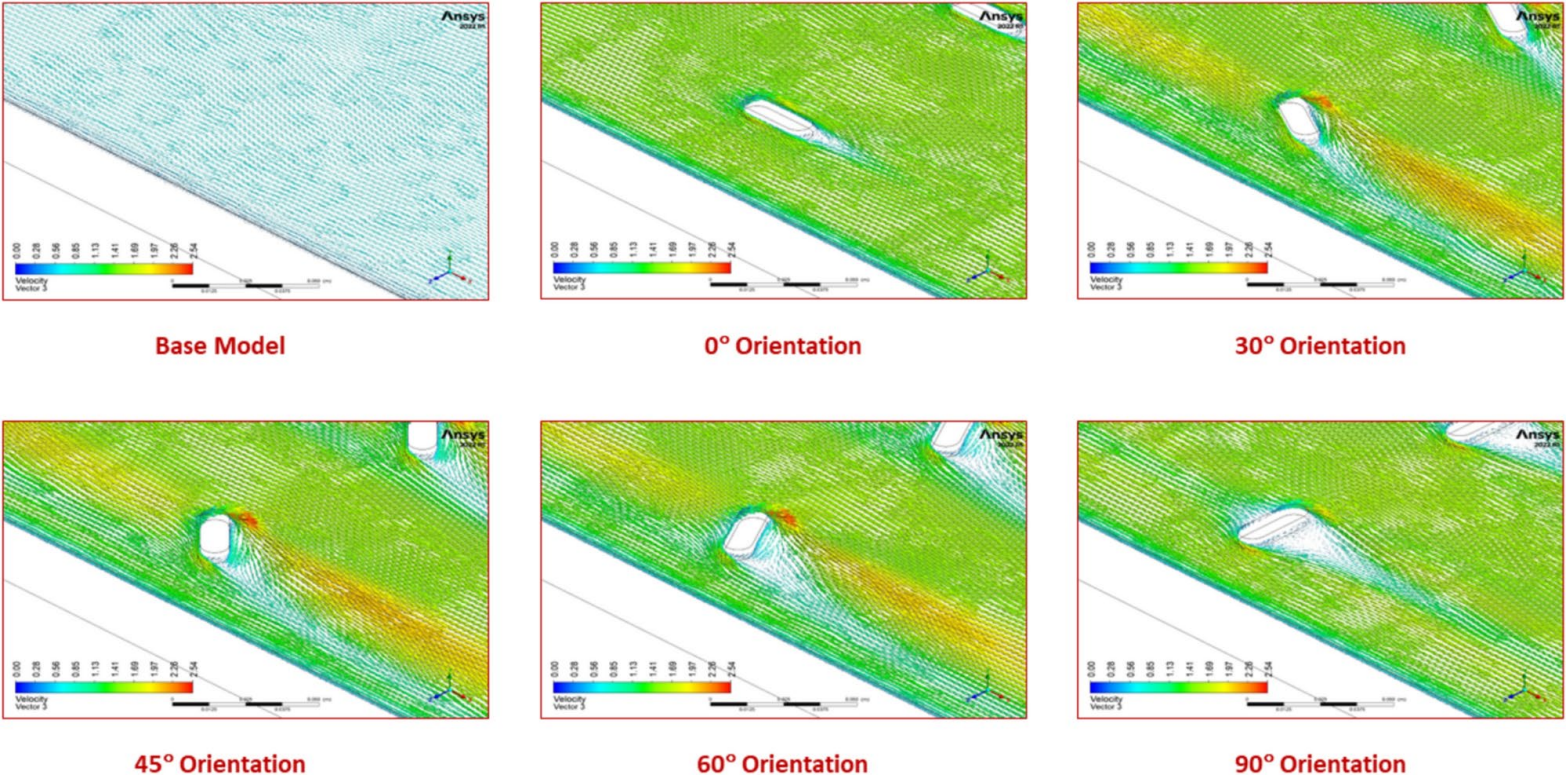
Velocity vector plots for various orientation angles.

The friction factor value reduces with the increasing Re value for all the orientation angles. This is due to the higher flow velocity of the fluid. For the orientation angle of 60°, the friction factor appears to be relatively higher when compared with other orientation configurations. This arrangement offers the highest fluid flow resistance, resulting in a superior pressure drop and, as a result, the highest pumping power is needed. Air takes a sharp turn in the upstream side of this turbulator configuration to surpass the obstruction, thereby increasing the pumping power requirement as shown in the velocity vector plot (Fig. 15). The other configurations exhibit marginally lesser pressure drop due to a smoother flow turning action near the turbulator.

#### Effect on TEF

**Fig. 16:**
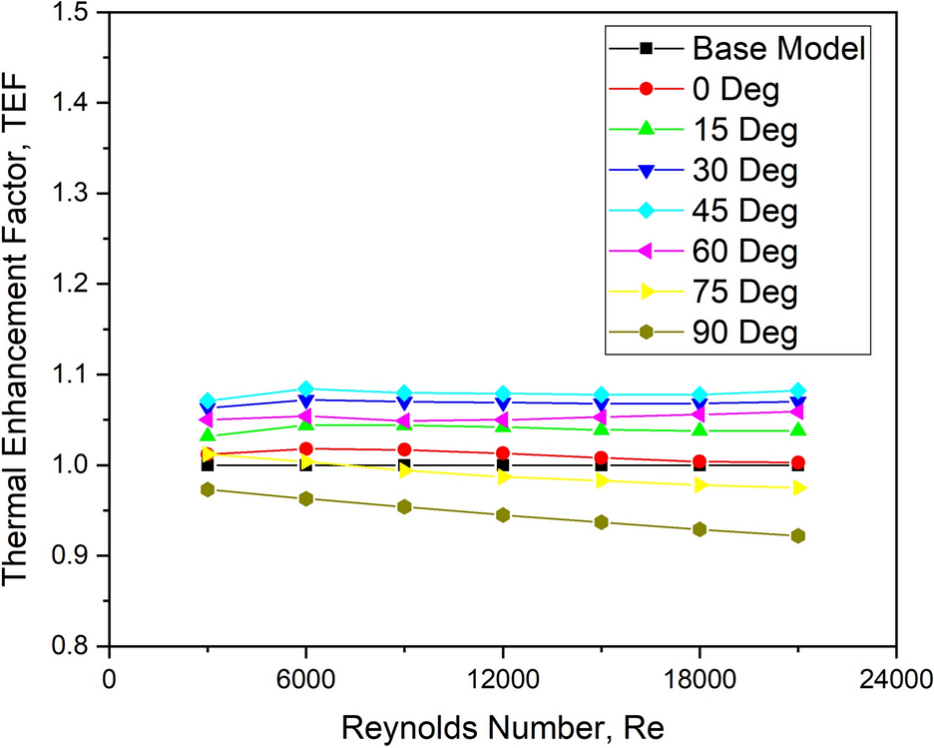
Variation of TEF with Re for various orientation angles.

The variation of TEF for various turbulator orientation angles is shown in Fig. 16. There is a slight increase in TEF at lower Reynolds numbers, followed by a reduction as the Re increases. The maximum value is observed at flow Reynolds number 6000.

Specifically, an orientation angle of 45° has a higher TEF value. TEF appears to be higher for this arrangement with a higher Nu and a marginally lower friction factor.

### Longitudinal Pitch variation

This section quantitatively analyses a solar air heater with capsule-shaped turbulators arranged with an optimized orientation angle. A two-row turbulator configuration with an optimized orientation angle of 45° and turbulator height ratio of 0.005 is used in this investigation. The longitudinal pitch ratio ranges from 0.025 to 0.25.

#### Effect on Nusselt number

**Fig. 17:**
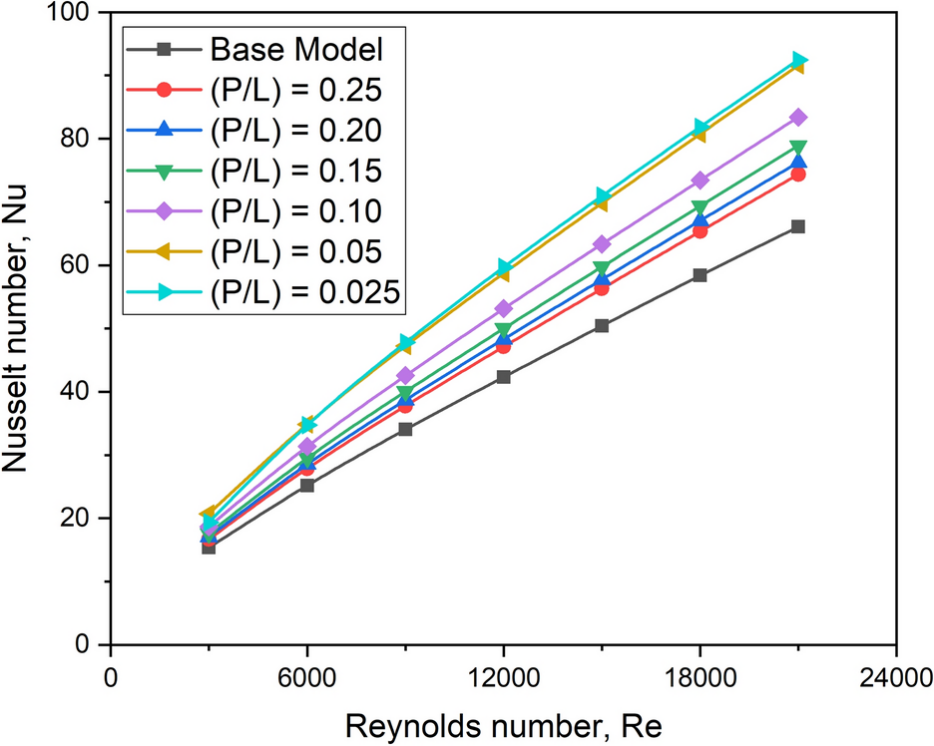
Nu versus Re for various longitudinal pitch ratios.

Figure 17 compares the Nusselt number for various longitudinal pitch ratio values. A turbulator at any given pitch improves the Nu unconditionally for all values of the Reynolds number, as the turbulator adds turbulence to improve convective heat transfer.

A wider pitch gap minimizes heat transfer because there are fewer turbulators on the absorber plate, reducing the influence of turbulators on heat transfer. As a result, a longitudinal pitch ratio of 0.25 produces a lower Nusselt number when compared to other longitudinal pitch ratio values used in the study.

**Fig. 18:**
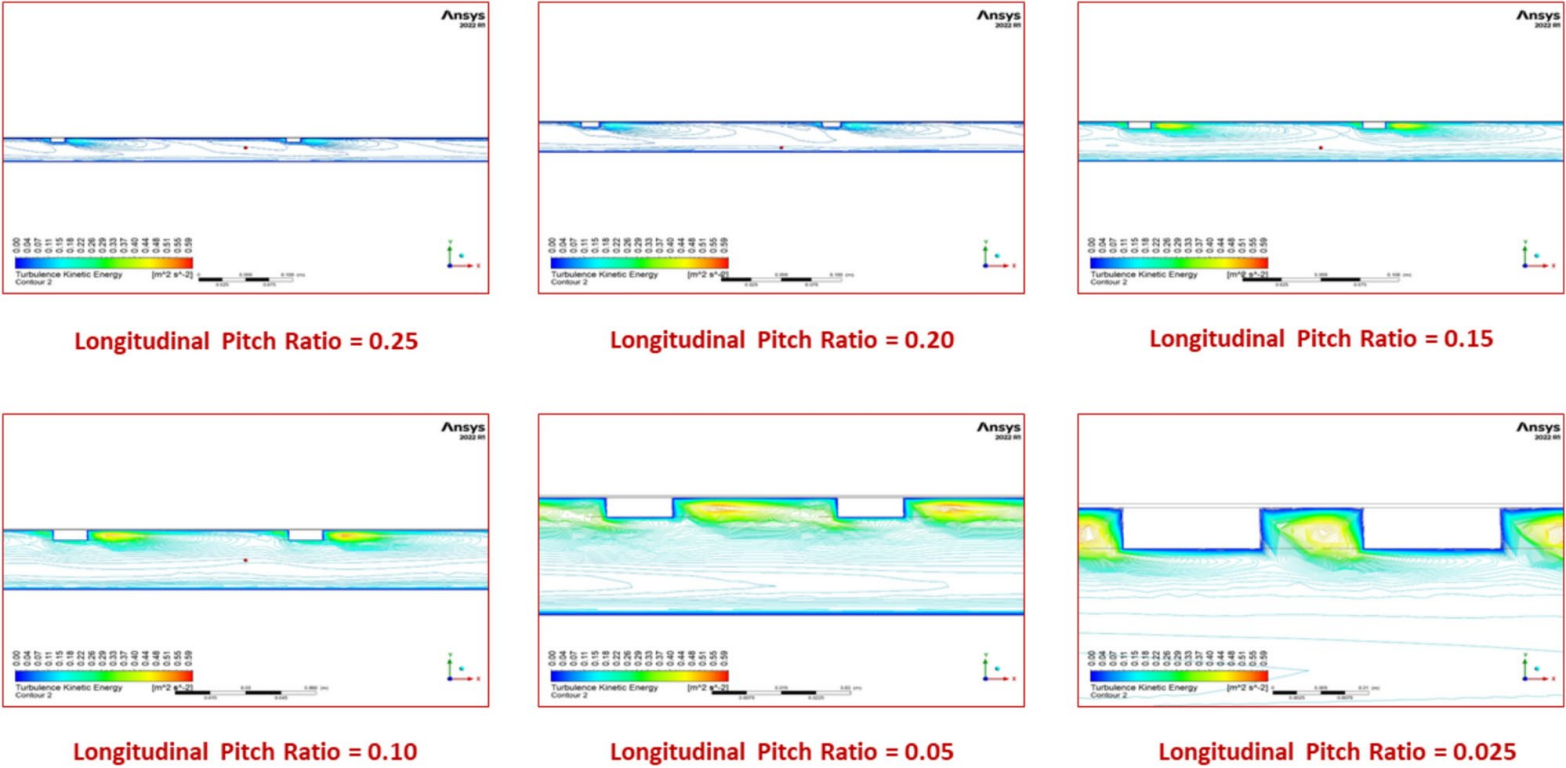
Contour plot of turbulent kinetic energy for various longitudinal pitch ratios.

The lower the longitudinal pitch, the better the convective heat transfer rate, which could be attributed to a larger number of oriented capsule-shaped turbulators with smaller pitch values, which results in more swirl motion for air near the inter-turbulator area, enabling enhanced heat transfer. The diagram shows a longitudinal pitch ratio of 0.025, which results in a relatively higher Nusselt number. This is because lower relative roughness pitch values induce more significant turbulence levels in the airflow, as shown in Fig. 18, which depicts the contours of turbulent kinetic energy. Furthermore, the Nusselt number increases monotonically with increasing Re for all tested conditions.

#### Effect on friction factor

**Fig. 19:**
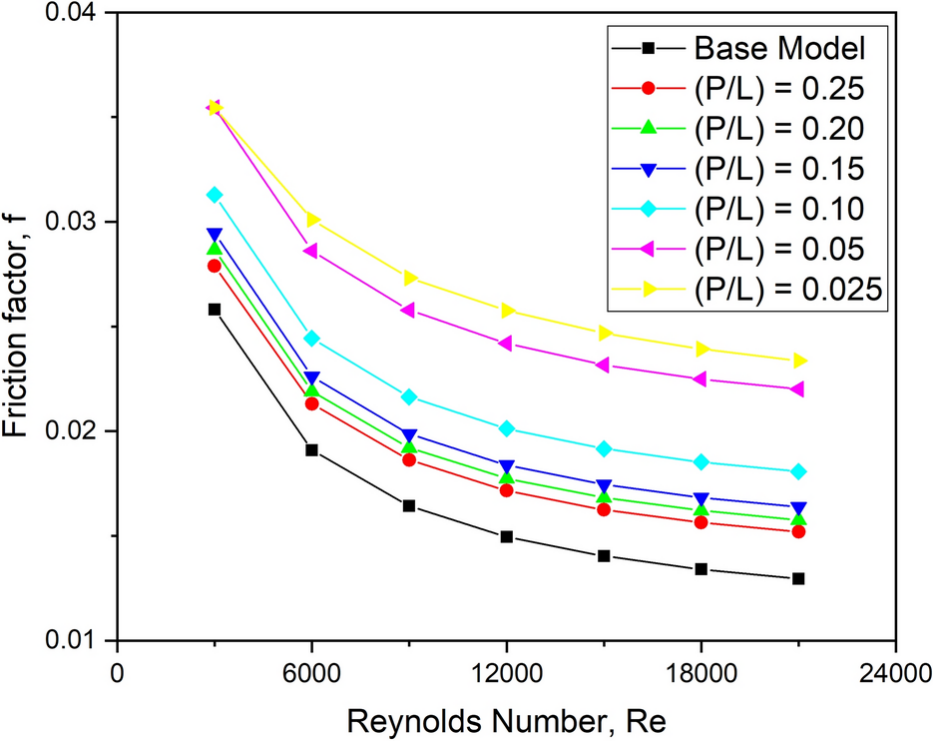
f versus Re for various longitudinal pitch ratios.

Figure 19 shows how the friction factor varies with the Reynolds number for different values of longitudinal pitch ratio. It can be shown that when the Reynolds number increases, the friction factor decreases.

Because of the higher flow velocity of the fluid, the friction factor value decreases with increasing Re value for all longitudinal pitch ratios. Compared to other longitudinal pitch configurations, the friction factor is comparatively larger for the longitudinal pitch ratio of 0.025. This configuration provides a significantly higher resistance to fluid flow, resulting in a more considerable pressure drop and, as a result, the most pumping power required. The blockage on the upstream side of this design increases the required pumping power. As the pitch ratio grows, the instances of turbulators in each row decrease, resulting in an inferior pressure drop.

#### Effect on TEF

**Fig. 20:**
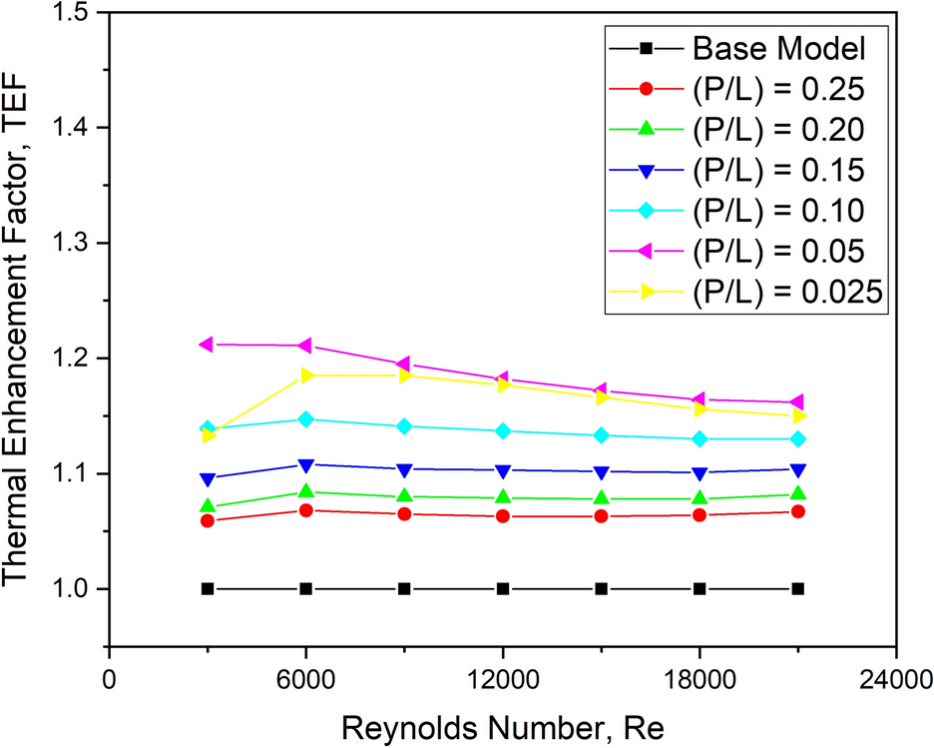
TEF versus Re for various longitudinal pitch ratios.

To understand the overall performance of a solar heat heater compared to that of the base model, TEF has been shown in Fig. 20. The graph displays the variation of TEF for several longitudinal pitch ratio values.

It is observed that when the Re rises, the TEF decreases. The maximum TEF value is seen for flows with Reynolds numbers of 6000. For all flow Reynolds numbers, the longitudinal pitch ratio of 0.05 indicates a considerably more significant value of TEF. TEF appears to be higher for this setup with a significantly larger Nu and marginal value of friction factor.

### Variation in turbulator height

The improvement in the characteristics of a solar air heater outfitted with capsule-shaped turbulators arranged with optimized orientation angle and optimized longitudinal pitch ratio is numerically examined in this section. A two-row turbulator configuration with an optimized orientation angle of 45° and an optimized longitudinal pitch ratio of 0.05 is used in this investigation. The turbulator height ratio ranges from 0.004 to 0.008.

#### Effect on Nusselt number

**Fig. 21:**
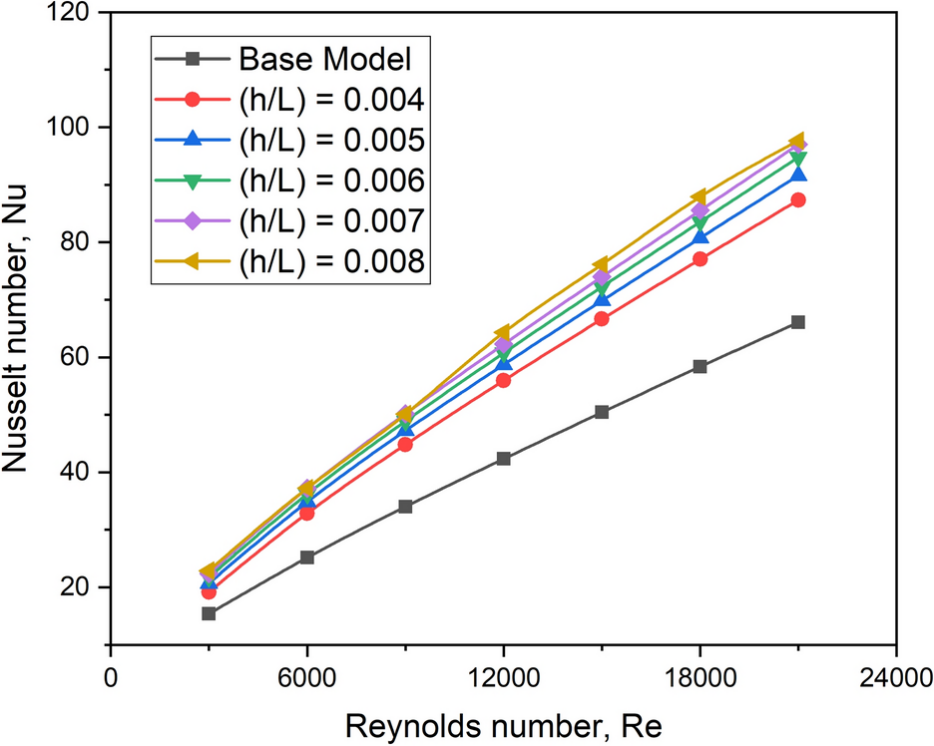
Nu versus Re for various values of turbulator height ratio.

Figure 21 compares the Nusselt number for various turbulator height ratio values to the flow Reynolds number. A turbulator at all of the chosen height ratios enhances the Nusselt number for all Reynolds number values, as the turbulator adds turbulence to the heat transmission. Compared to alternative configurations, including the base model, a height ratio of 0.008 is optimal among the chosen height ratio values, yielding a considerably higher Nusselt number.

**Fig. 22:**
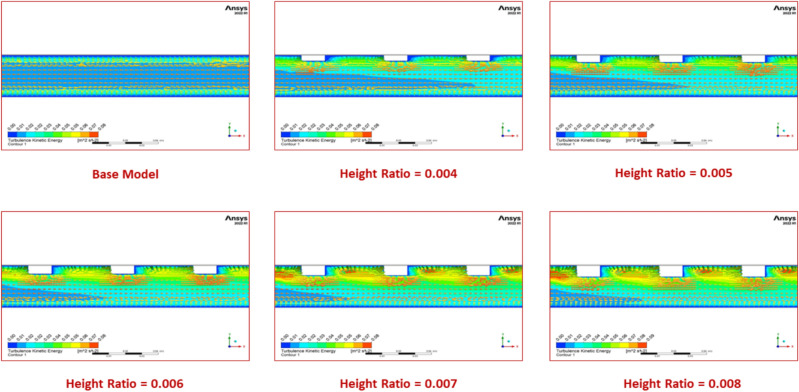
Contour plots of turbulent kinetic energy for various values of turbulator height ratio.

In this case, with a height ratio of 0.008, the turbulator generates increased turbulence on the downstream side as well as on the turbulator’s bottom, boosting flow mixing as seen in contour plots (Fig. 22). As a result, this design will produce a higher convective heat transfer coefficient.

#### Effect on friction factor

**Fig. 23:**
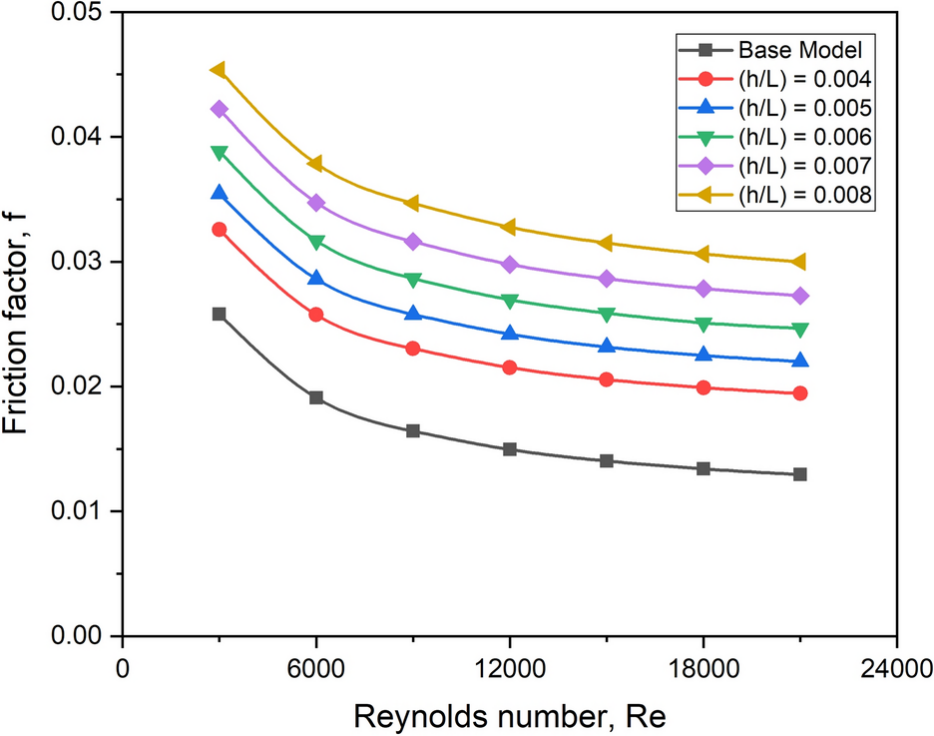
f versus Re for various values of turbulator height ratio.

Friction increases with increasing turbulator height, regardless of the Reynolds number. Figure 23 depicts the fluctuation of the friction factor concerning the turbulator height and flow Reynolds number.

As the turbulator height increases, it generates extra turbulence in its surroundings, resulting in sudden flow halting. As a result of the partial flow blockage, the height ratio of 0.008 experiences a more considerable decrease in pressure.

#### Effect on TEF

**Fig. 24:**
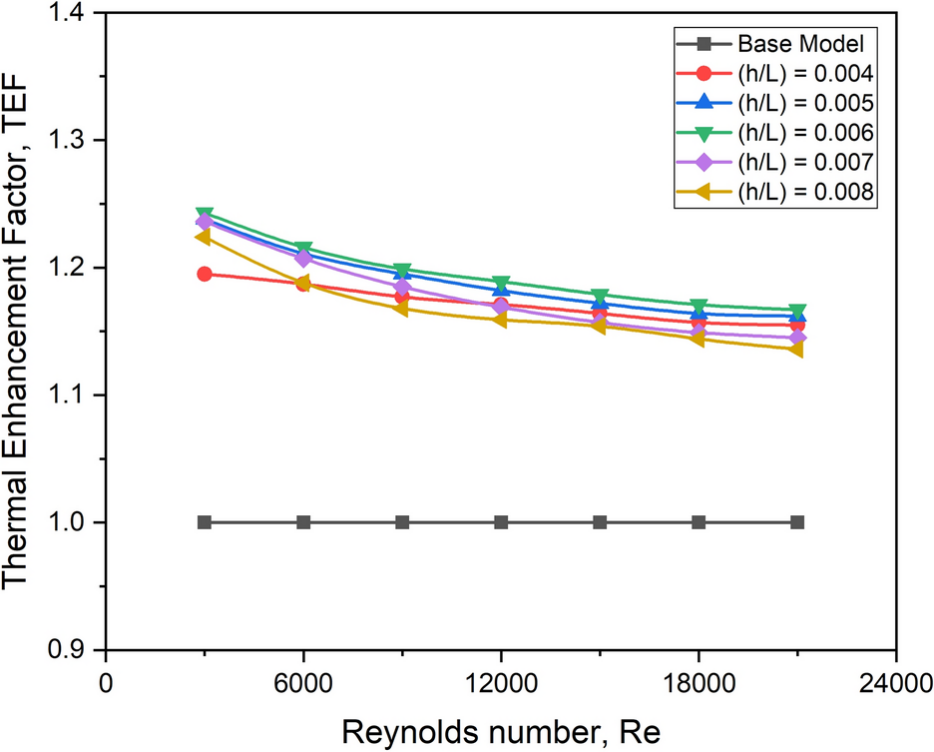
TEF versus Re for various values of turbulator height ratio.

The graph in Fig. 24 depicts the relationship between TEF and Reynolds number for different turbulator height ratio values. TEF is observed to have a restricted incremental trend at low mass flow rates and then declines as the mass flow rate or Re increases.

TEF is shown to be higher in all configurations for a turbulator height ratio of 0.006, especially on the higher Reynolds number side. It is because there is optimal extra turbulence, which promotes convective heat transfer and dominates the improvement in friction factor. The combined impact is to improve overall TEF performance.

### Variation in turbulator row arrangement

This section examines the solar air heater fitted with capsule-shaped turbulators arranged with an optimized orientation angle, optimized longitudinal pitch ratio, and optimized height ratio through a whole-field CFD analysis. This study uses an optimal orientation angle of 45°, an optimized longitudinal pitch ratio of 0.05, and an optimized height ratio of 0.006. In the transverse direction, turbulators are organized in single-row, two-row, and three-row designs.

#### Effect on Nusselt number

**Fig. 25:**
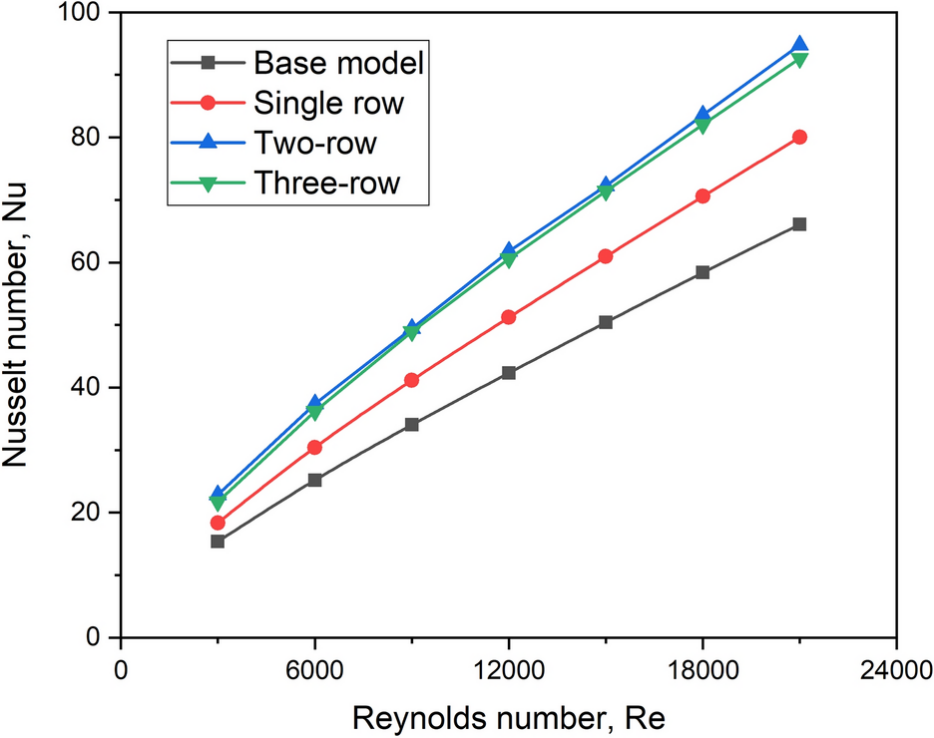
Nu versus Re for various row arrangements.

Figure 25 compares the Nusselt number for various turbulator arrangements for different cases of Re. It is seen that the presence of turbulators improves the heat transfer characteristics at all the Reynolds numbers, irrespective of rows of turbulators.

**Fig. 26:**
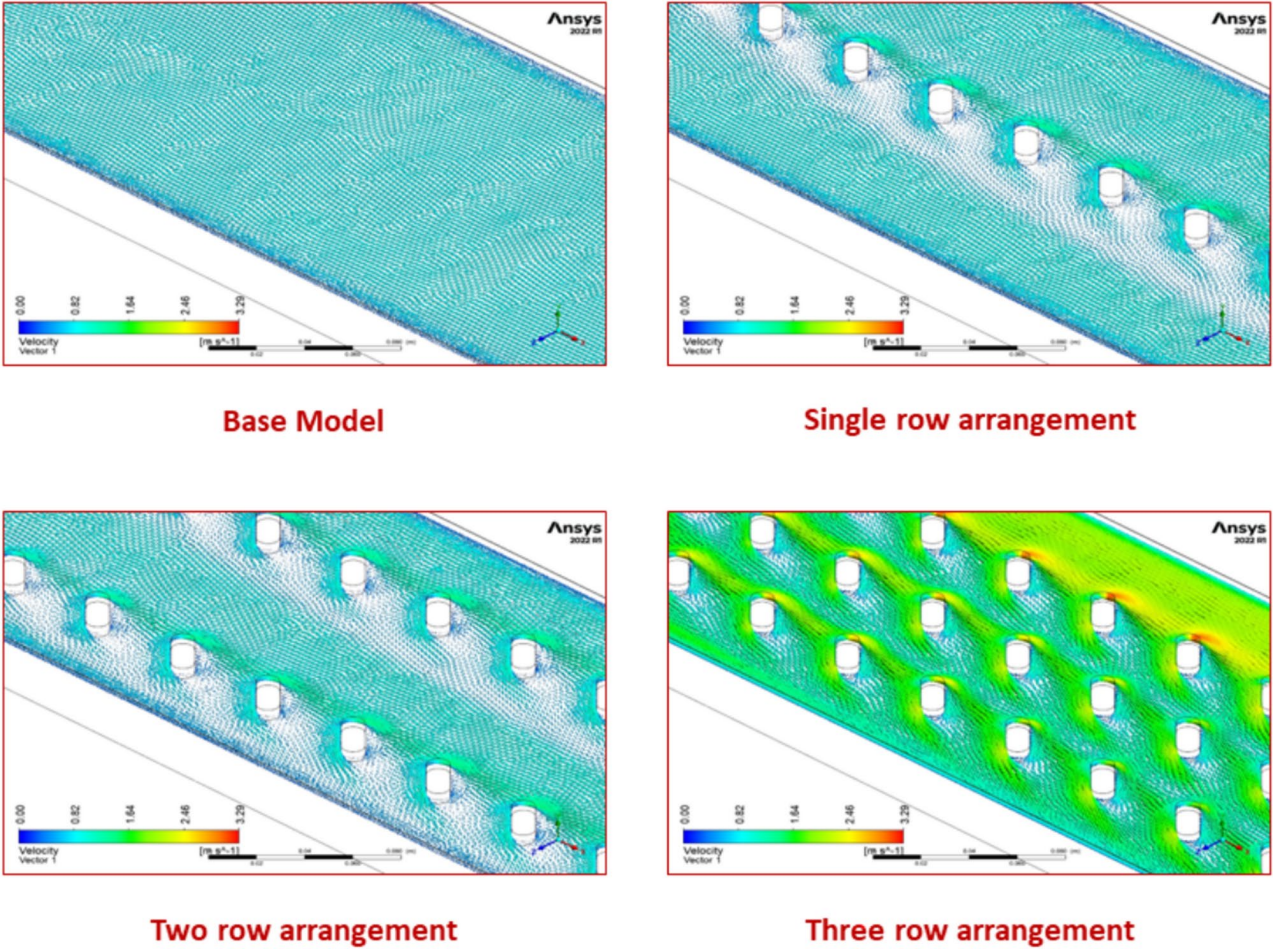
Velocity vector plots for various row arrangements.

It is found that a two-row arrangement yields a better Nu for all the flow Reynolds numbers chosen for the study. This is because, with lower Reynolds numbers, air travels through the area of each turbulator at a lower velocity. This increases the heat exchange time, hence Nu. The flow outruns the turbulator at higher Reynolds numbers with marginally increased speed, resulting in effective turbulence. This results in more significant convective heat transfer and, as a result, a larger Nusselt number. These events are well-supported by velocity vector plots, as illustrated in Fig. 26.

#### Effect on friction factor

**Fig. 27:**
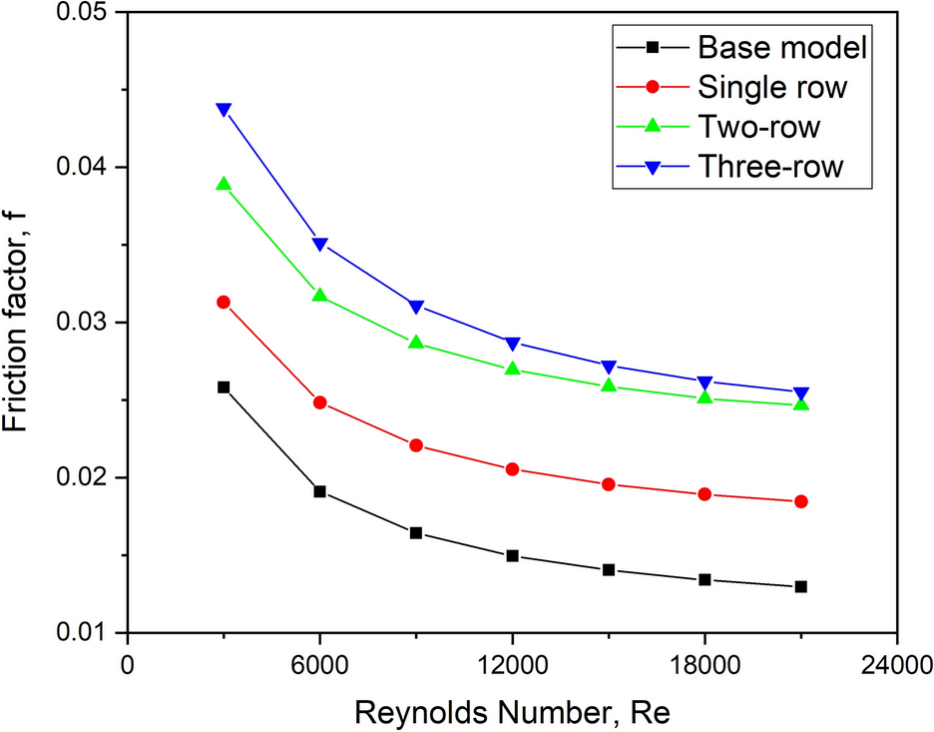
f versus Re for various row arrangements.

As the instances of turbulators are proportional to the rows of arrangement, it is well-known that adding a higher number of turbulators results in a relatively higher friction factor. The graph shown in Fig. 27 depicts its variation for various row configurations of turbulators.

As the number of rows of turbulators increases, the fluid must overcome the surface of the turbulator, which generates a more significant pressure drop towards the upstream side of the succeeding turbulator. As a result, the configuration with more turbulators produces a more considerable pressure drop and, thus, a higher friction factor. As a result, for all flow Reynolds number circumstances, a three-row configuration suffers from a larger value of friction factor.

#### Effect on TEF

**Fig. 28:**
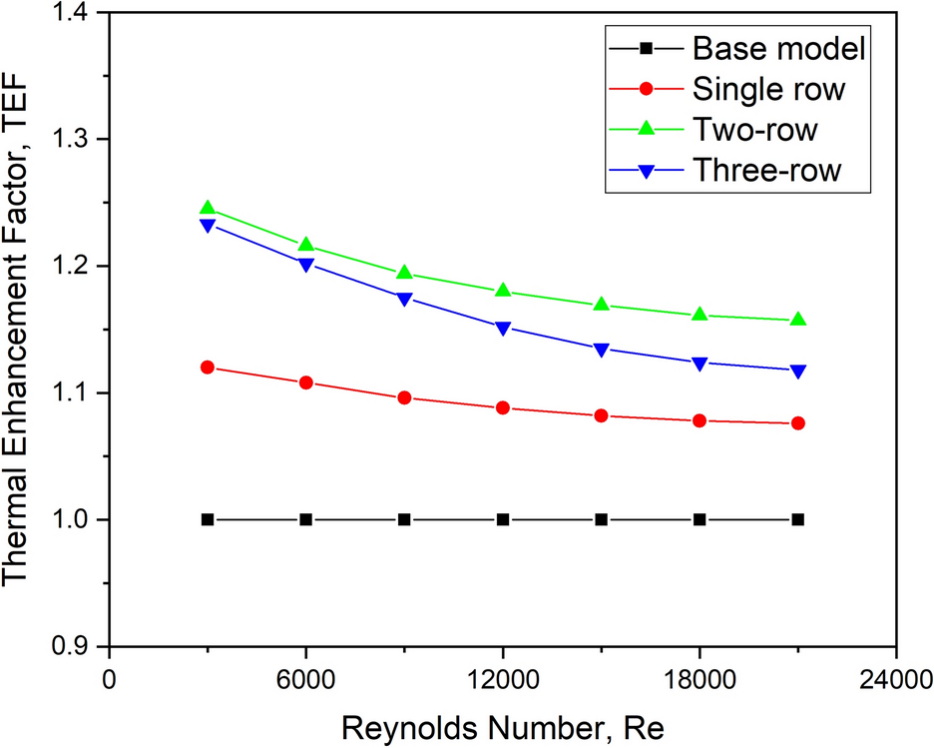
TEF versus Re for various row arrangements.

For various turbulator row configuration patterns, the graph in Fig. 28 demonstrates the relationship between TEF and Reynolds number. TEF is seen to have a decreasing tendency as the flow Reynolds number increases for all row configurations chosen for the investigation.

TEF is higher in two-row designs for all the flow conditions. Because greater convective heat transfer dominates frictional losses at lower mass flow rates, the value of TEF improves. When the mass flow rate increases, the fluid bypasses the turbulator faster. As a result, the convection-dominated flow has a larger Nusselt number at higher flow Re. There is sufficient clearance space between successive turbulators in the transverse direction, and frictional losses are deemed minor. The combined effect improves overall TEF performance for two-row configurations.

Finally, a solar air heater equipped with an absorber plate containing a capsule-shaped turbulator of 6 mm height, oriented at 45° to the flow direction, arranged with a longitudinal pitch ratio of 0.05 and organized as a two-row arrangement is considered to be an optimized configuration and can be a design prescription for the next generation solar air heater.

### Comparison of base model and optimized model

#### Thermohydraulic performance parameter

It is critical to examine the effect of the Stanton number in addition to the Nusselt number because the Stanton number considers the fluid’s thermal heat capacity and the heat exchange between the air and the absorber plate.

**Fig. 29:**
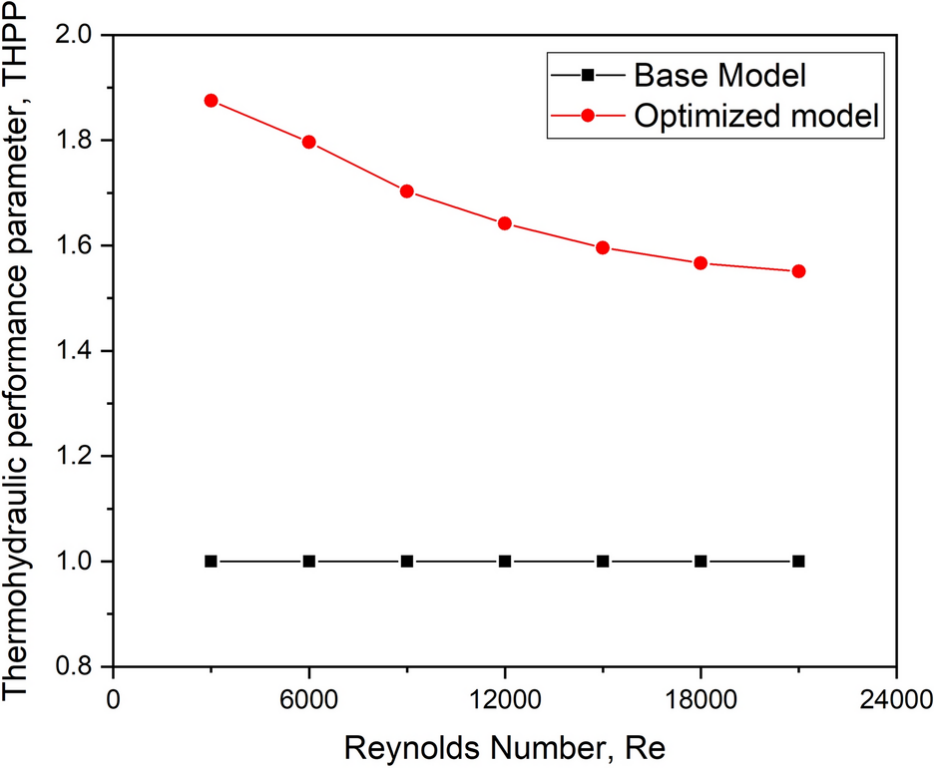
THPP versus Re for the base model and the optimized configuration.

The graph depicts the fluctuation of THPP with flow Reynolds number for the optimized turbulator configuration and the base model configuration without the turbulator. This pattern resembles the TEF plot seen in Fig. 29. As a result, this figure contributes to the conclusion that THPP is consistent with TEF, with the optimized turbulator arrangement of 6 mm height, oriented at 45° to the flow direction, arranged with a longitudinal pitch ratio of 0.05 and organized as a two-row arrangement for all flow Reynolds numbers.

#### Absorber plate temperature index

**Fig. 30:**
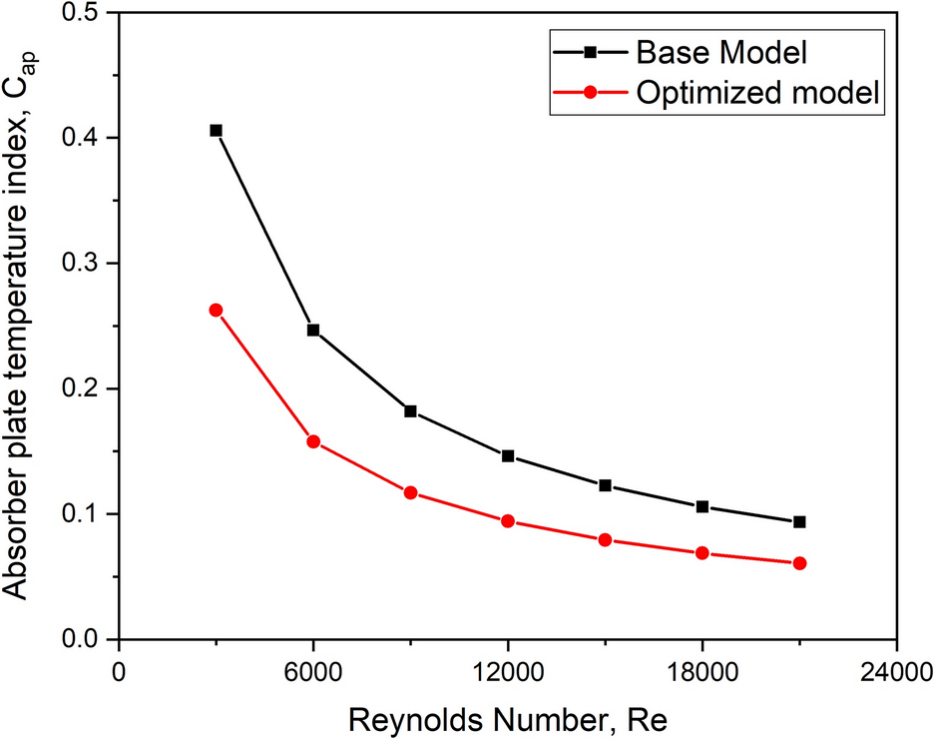
Absorber plate temperature index versus Re for the base model and the optimized configuration.

The influence of the absorber plate temperature index at various mass flow rates is depicted in Fig. 30 for the base model and the optimized turbulator design. Within the Reynolds number range used in the analysis, the base model absorber plate exhibited the highest temperature index compared to the optimized model.

This may be due to turbulators increasing the active area, which improves energy transfer to the air stream. This allows more heat to be transported away, allowing the absorber to remain relatively at a lower temperature.

#### Exergy efficiency

**Fig. 31:**
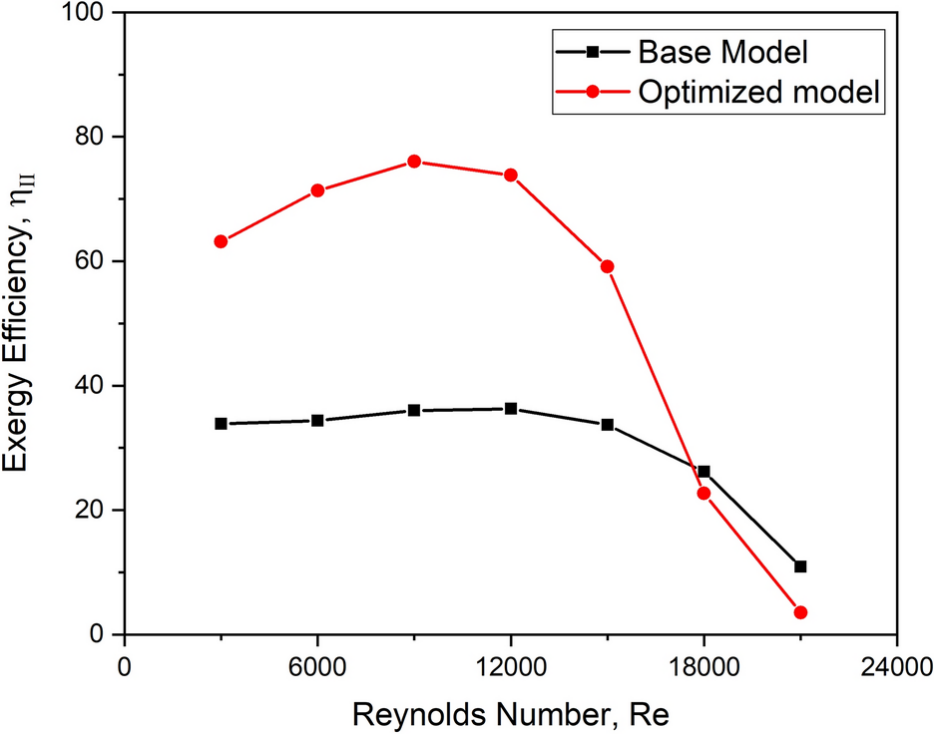
Exergy efficiency versus Re for the base model and the optimized configuration.

Exergy analysis is an effective thermodynamic-based technique for determining irreversible loss forms, locations, and amounts. The disparity in exergy efficiency with the Reynolds number for the base model and optimized configurations is shown in Fig. 31.

It can be shown that the exergy efficiency peaks at a flow Reynolds number of 9000 and subsequently drops as the Re increases. At this Reynolds number, the exergy efficiency of the optimized model is 52.63% more than that of the base model.

**Fig. 32:**
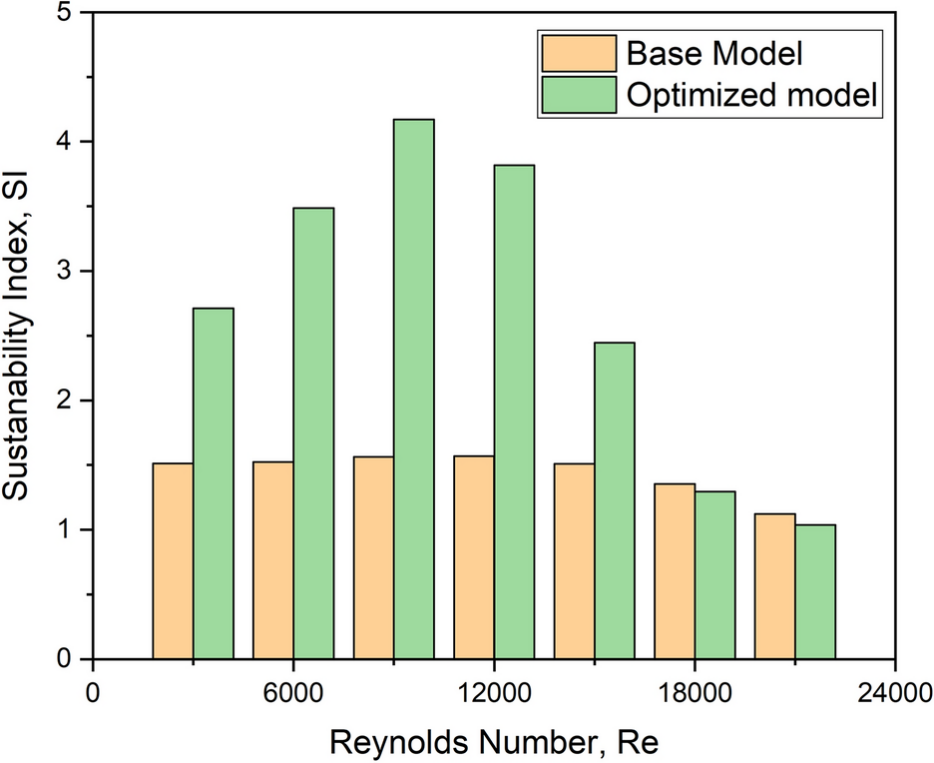
Sustainability index versus Re for the base model and the optimized configuration.

This phenomenon is entirely dependent on the fluid’s exergy degradation. As a result, the collector collects more solar heat effectively with turbulators, especially at lower Reynolds numbers within 9000. This is corroborated by comparing the sustainability index for the optimized configuration and the base model, as shown in Fig. 32.

Based on these findings, it can be inferred that the optimized turbulator configuration is more effective up to a flow Reynolds number of 9000.

### Comparison of present investigation with previous works

Table 6 provides a thorough comparison of the THPP of the current investigation with that of several turbulence promoters documented in earlier research. Because rib designs create secondary vortices that travel down the airstream instead of being stationary, as is the case with transverse ribs, the comparison shows that the THPP is often larger for rib designs. The THPP is enhanced due to the improved flow mixing between the hot and cold airstreams close to the heated absorber plate.

**Table 6:** Comparison of the present study with available literature.

Type of turbulator	Parameters chosen	THPP
Multiple V-shaped ribs^41^	Re: 9000–21,000 Rib angle: 10° Relative pitch: 10	2.4
Spherical-shaped turbulence promoters^42^	Re: 3000–14,000 Attack angle: 30° -60° Relative gap: 0.045	2.01
Perforated-winglet-type vortex generator^43^	Re: 4000–25,000 Winglet height = 0.2–0.48 Hole diameter: 1 mm–7 mm	2.01
Hybrid transverse semi-circular and triangular Rib^44^	Re:12,000–50,000, Normalized pitch: 6.6– 53.3 Aspect ratio: 4	1.34
Discrete symmetrical arc rib^45^	Re: 3000–14,000 Gap width ratio: 2–5 Rib height ratio: 0.045 relative pitch: 10	1.68
Present study	Re: 3000–24,000 Orientation angle: 0°–90° Longitudinal pitch: 25 mm–250 mm Turbulator height: 4 mm–8 mm	1.88

## Conclusion

A detailed study was undertaken to analyse the performance enhancement of adding capsule-shaped turbulators on a typical flat plate solar air heater; the conclusions of the study can be summarised as follows:

1. It is discovered that the Nu is significantly affected by the presence of a turbulator for energy transfer and the flow behaviour, which may be unique to each design.
2. For the whole range of flow Reynolds numbers, the thermal enhancement factor of turbulator design with a 45° orientation angle is determined to be maximum.
3. The collector performs best for a longitudinal pitch ratio 0.05 under all flow Reynolds number conditions.
4. The thermal enhancement factor was the highest for the turbulator height ratio of 0.006 at all mass flow rates studied.
5. The two-row turbulator configuration considerably upsurges the localized Nu over the turbulators due to the creation of complicated vortex flows at each turbulator’s downstream end, resulting in a higher performance.
6. The thermohydraulic performance parameter follows the same trend as the thermal enhancement factor, with the optimized design with two rows having the highest value of 1.88.
7. The optimized model’s exergy efficiency is 52.63% more than the base model at the flow Reynolds number 9000.

## Data Availability

Data that support the findings of this study are available from the corresponding author, Dr Shiva Kumar, upon reasonable request.

## List of symbols

Nu: Nusselt number
h_c_: Convective heat transfer coefficient (W/m^2^K)
L_c_: Characteristic length (m)
K: Thermal conductivity of air (W/mK)
f: Friction factor
$\Delta p$: Pressure drop (Pa)
$\rho$: Air density (kg/m^3^)
L: Length of test section (m)
V: Velocity of air (m/s)
C_ap_: Absorber plate temperature index
T_Top_: Temperature of absorber surface (K)
TEF: Thermal enhancement factor
T_1_: Air inlet temperature (K)
THPP: Thermo-hydraulic performance parameter
S_t_: Stanton number
$$\eta_{II}$$: Exergy efficiency
Ex: Exergy rate (W)
$\dot{m}$: Mass flow rate (kg/s)
$\Delta h$: Enthalpy change (J/kg)
$\Delta s$: Change in entropy (J/kg K)
SI: Sustainability index
W: Width of the absorber plate (m)
H: Height of the air duct (m)
SAH: Solar air heater
CFD: Computational fluid dynamics
p: Static pressure (Pa)
$$\alpha$$: Orientation angle (°)
RNG: Re-normalized group
Pr: Prandtl number
k: Turbulent kinetic energy (J/kg)

## Suffix

1: Inlet to air duct
2: Exit from the air duct
t: Turbulator model
s: Base model
